# Protective Effects of Korean Herbal Remedy against Airway Inflammation in an Allergic Asthma by Suppressing Eosinophil Recruitment and Infiltration in Lung

**DOI:** 10.3390/antiox10010006

**Published:** 2020-12-23

**Authors:** Soyon Yoon, Seokcheon Song, Jae Woo Shin, Sini Kang, Hye Young Kim, Hyun Ju You

**Affiliations:** 1 Department of Environmental Health Sciences, Graduate School of Public Health, Seoul National University, Seoul 08826, Korea; alex.sy.yoon@gmail.com (S.Y.); ursa20@snu.ac.kr (S.S.); 2 Laboratory of Mucosal Immunology, Department of Biomedical Sciences, Seoul National University College of Medicine, Seoul 110799, Korea; sinsin0818@snu.ac.kr; 3 Department of Food and Nutrition, Research Institute of Human Ecology, Seoul National University, Seoul 08826, Korea; kangsini@snu.ac.kr; 4 Institute of Health and Environment, Graduate School of Public Health, Seoul National University, Seoul 08826, Korea

**Keywords:** phytochemical, allergic asthma, ovalbumin, innate lymphoid cell

## Abstract

The increasing prevalence of allergic asthma has become the world’s major health issue. Current treatments for allergic asthma focus on treating symptoms, while permanent cures still remain undiscovered. In this study, we investigated the effect of Korean traditional herbal remedy, Pyunkang-tang (PGT)—composed of six plants—on asthma alleviation in a mouse model. The PGT mixture was orally gavaged to mice (PM group, 20 mg/mouse/day) from 7 days before sensitization with ovalbumin (OVA) (day −7). On day 0 and day 14, mice from OVA-control (*n* = 9) and PM group (*n* = 8) were sensitized with OVA and alum through intraperitoneal injection. On days 18~20, OVA was challenged to mice through nasal injection and sacrificed next day. Cell profile in lung tissue was analyzed by flow cytometry and RT-qPCR analysis, and the number of eosinophils and expression of *siglec-F* were significantly reduced in the PM group. Lung tissue was examined with hematoxylin and eosin (H&E) and Alcian blue/periodic acid–Schiff (AB-PAS) staining. Noticeably reduced eosinophil infiltration around bronchioles was displayed in the PM group compared to the OVA-control group. Furthermore, PGT-treated mice showed a significant reduction in IL-13 and a mild reduction in IL-5 in lungs. A decreasing tendency of IL-5/13 (+) CD4+ T cells and IL-13(+) innate lymphoid cells (ILCs) and a significant reduction in IL5(+) ILCs were also observed. When treating PGT on murine lung epithelial cells stimulated by papain, there was a significant reduction in IL-33 mRNA expression levels. Taken together, oral delivery of PGT successfully alleviated asthmatic responses provoked by OVA in a mouse model and could lead to novel therapies for allergic asthma.

## 1. Introduction

Asthma is a term widely used to describe a chronic inflammatory pulmonary disease characterized by various and recurrent symptoms which can have a wide extension of severity. Such symptoms include wheezing, coughing, reversible airflow obstruction and bronchospasm. Worldwide, about 339 million people suffer from asthma [1], and it contributes 13.8 million disability-adjusted life years (DALYs) annually [2]. Socio-economically, asthma is one of the major sources of global economic burden especially due to its high incidence, prevalence and the chronic nature [2,3,4].

Allergic asthma is the most common form of asthma that is often characterized by eosinophilic airway inflammation [5,6,7]. Type 2 helper cell (Th2) responses mainly drive the pathogenesis of the disease [8,9,10]; it is characterized by interleukin IL-5 and IL-13 dependent lung eosinophilia, airway hyperresponsiveness (AHR), goblet cell hyperplasia, and IL-4 dependent immunoglobulin E (IgE) elevation in serum [7,8,9,10,11,12,13,14]. Current treatments for allergic asthma mainly focus on attenuating allergic responses upon allergen exposure. Inhaled steroids such as corticosteroids [15,16,17], monoclonal anti-IgE antibody [18,19,20,21] and bronchodilators [22,23] are popular treatments, but their efficacy is restricted to reducing the symptoms without curing the disease [14,17]. Furthermore, these treatments are not effective for all patients [24,25]; there also are potential side effects [26,27,28,29]. As a result, there is an increasing demand for novel treatment strategies that are not restricted to reducing allergic responses, but rather that focus on attenuating the earlier stages of pathogenesis [14].

Complementary and alternative medicine (CAM) is termed, any medical and health care products or practices which are distinguished from standard medicines. Herbal remedies are one of the most popular methods among CAM to treat asthma, of which the use has increased dramatically worldwide in the past decades. Plant-based traditional medicine is an excellent example of herbal remedies, and there are increasing numbers of studies being conducted to prove the efficacy of traditional medicine on allergic asthma. More specifically, there is growing evidence that certain phytochemicals from traditional medicine have a significant effect on a variety of chronic diseases, including asthma.

*Lonicerae Flos* (LF)*, Liriopis Tuber* (LT)*, Adenophorae Radix* (AR)*, Xanthii Fructus* (XF)*, Selaginellae Herba* (SH)*,* and *Rehmanniae Radix Preparata* (RRP) are parts of plant species (i.e., flowers, roots, fruits and leaves) which are common oriental medicinal herbs in Korea, Japan, and China. To be specific, LF can be considered as a source of natural antioxidants, which may be due to its chlorogenic acid component [30]. It has latent-heat-clearing, antipyretic, detoxicant, and anti-inflammatory effects [31]. LT has shown to have the anti-inflammatory and anti-asthmatic effect [32]. AR modulates the activity of mast cells and respiratory mucous generation, and further exerts antitussive, expectorant, anti-asthmatic and anti-inflammatory effects [33,34]. Additionally, XF displays antioxidant, antinociceptive, and anti-inflammatory activities [35]. SH has been reported to relieve phlegm and asthma symptoms [36]. RRP has been widely utilized as an anti-inflammatory treatment [37].

A Korean traditional remedy called Pyunkang-tang (PGT), consisting of the six herbs mentioned above, has been used as traditional herbal medicine for managing the inflammatory hypersecretion of airway mucus present in chronic obstructive pulmonary disease (COPD), pneumonitis, tonsilitis, as well as pulmonary fibrosis for years [37]. PGT decreased the release of inflammatory cytokines in bronchoalveolar lavage fluid and mucus secretion in a rat COPD-like pulmonary inflammation model induced by mixture of cigarette smoke extract and sulfur dioxide [38]. Moreover, PGT showed alleviation of symptoms in patients suffering from asthma, atopic dermatitis, rhinitis, and food allergy [39].

In this study, we aimed to demonstrate the efficacy of the PGT mixture on alleviating ovalbumin (OVA)-induced allergic asthma in mice and to investigate the mechanism underlying reduced inflammation. Daily oral dosing with the PGT mixture abrogated the disease pathogenesis by decreasing infiltration of eosinophils in the lung. PGT reduced the Th2 response while not elevating the Th1 response. With its potential to modulate various immune responses, PGT can be further tested to be a novel therapeutic for allergic asthma.

## 2. Materials and Methods

### 2.1. Preparation of Pyunkang-Tang (PGT) Mixture

PGT is a water extract of six herbs (Table 1). The voucher specimens were deposited at the Herbarium of the College of Pharmacy, Kyung Hee University (Seoul, Korea). The six herbs were purchased from Dae-won-dang Oriental Drug Store (Seoul, Korea). The preparation of PGT water extract was conducted as previously described [37]. The 40 g of six herbal mixture were soaked in 500 mL of double-distilled deionized water and decocted for 150 min at 100 °C in a closed system. The extract was filtered through sterile gauze, concentrated in a rotary vacuum evaporator, lyophilized and stored at −70 °C until tested. Approximately 615 mg of lyophilized PGT extract mixture was prepared from 40 g of dried herbs (yield: 1.54%). To develop the oral treatment of PGT to mice, the lyophilized PGT extract was prepared in phosphate saline buffer (PBS) at a final concentration of 100 mg/mL.

### 2.2. Non-Targeted Metabolites Analysis of PGT by UPLC/MS

Lyophilized powder of PGT was dissolved in 99% methanol at a final concentration of 1 mg/mL. The subsequent solution was filter-sterilized using 0.22 µm syringe filter. To investigate the exploratory and non-targeted metabolite profile of PGT extract, we applied the UPLC/MS method [40,41] to phytochemicals and water-soluble components in PGT. Three PGT samples were analysed by qualitative metabolites profiling. An Acquity ultra-perforce liquid chromatography (UPLC, Waters, Milford, MA, USA) was used with a C18 BSH (130 Å, 1.7 µm, 2.1 mm × 100 mm) column (Waters). Preparation was conducted using solvent A (0.1% FA in 100% high-grade water) and solvent B (0.1% FA in 100% methanol). The flow rate was 0.176 mL/min starting with 100% solvent A for 1 min. Afterwards, a linear gradient was run in 59 min to 100% solvent B. The post run was 5 min with 100% solvent B and additional 10 min with 100% solvent A for re-equilibration. Mass spectrometry (MS) was performed using a Synapt G2-S mass instrument (Waters) equipped with an electrospray ion (ESI) source operated in high-resolution mode (ESI+, positive) or (ESI−, negative) mode. All mass spectral data were collected in centroid mode using the MSe mode of operation The column temperature was 52 °C and the injection volume was 10 µL. The separation of peaks in the PGT extract was checked on a diode array detector. The total ion chromatogram (TIC) and the base peak chromatogram (BPI) were displayed by MS measurement. In this study, the MS was set to positive ESI mode with a scan range from 50–2000, because the nitrogen-containing compounds would be abundant in herbs and plants. The capillary voltage was 3 kV. The sampling cone was set to 30 and the extraction cone to 3.0. Source temperature was set to 120 °C and desolvation temperature to 450 °C. The flow of the cone gas was 10 L/h and of the desolvation gas 800 L/h. The instrument was calibrated before analysis with 0.5 mM sodium formate solution. The identification of selected components and data preprocessing was performed using MassLynx^TM^ V4.1 (Waters Corp., Milford, MA, USA) software, version 4.1.

### 2.3. Ovalbumin-Induced Allergic Asthma in a Mouse Model and PGT Mixture Treatment

Six weeks old female BALB/c mice were purchased from Orient Bio Inc. (Seongnam, Korea). Upon arrival, mice were randomly allocated to individually ventilated cages that have available portable water and food ad libitum. Mice were kept in a semi specific pathogen-free facility at Seoul National University (Seoul, Korea). To investigate the effect of PGT mixture, we used a mouse model of allergic asthma induced by ovalbumin (OVA) and aluminium hydroxide (alum) [42]. Mice were divided into three groups: saline-challenged PBS treatment group (naïve, No-OVA-PBS, *n* = 8), OVA-challenged PBS treatment group (vehicle control, OVA-PBS, *n* = 9) and OVA-challenged PGT mixture treatment group (OVA-PM, *n* = 8).

**Figure 1:**
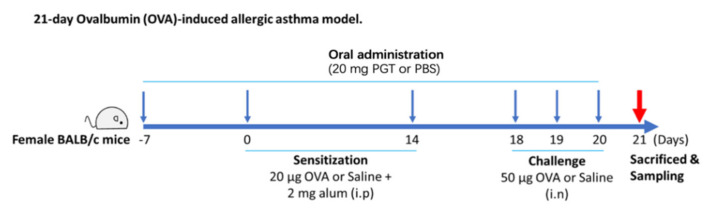
Experimental process diagram.

As shown in Figure 1, the PM group received daily oral gavage of 20 mg of PGT in 200 µL PBS from day −7 to day 20. The dosage was calculated based on the usual dosage for human in Korean Medicine Clinic. At the first week (day 0) and the third week (day 14) after oral administration, mice from OVA-control (*n* = 9) and PM group (*n* = 8) were sensitized with OVA and alum. Sensitization was conducted through intraperitoneal injection of 20 µg ovalbumin (OVA) (Sigma Aldrich, St. Louis, MO, USA) absorbed in 2 µg of Imject^®^ Alum (Thermo Fisher Scientific, Waltham, MA, USA) on days 0 and 14. On days 18, 19 and 20, mice were challenged intranasally with 50 µg of OVA. On day 21, animals were sacrificed, followed by collection of blood and lung tissue for further analysis. All experiments were in accordance with the Seoul National University Institutional Animal Care and Use Committee (IACUC) guidelines (No. SNU-170116-4). Mice were sacrificed at the end of experimentation by isoflurane (Hana Pharm Co., Ltd., Seoul, Korea) anesthesia and cervical dislocation.

### 2.4. Isolation of Lung and Histological Analysis

Lungs were used for histopathological examination as previously described [43]. Briefly, twenty-four hours after final OVA challenge, right middle lobes were removed by dissection and fixed in 10% neutral buffered formalin (Sigma Aldrich, St. Louis, MO, USA). The fixed tissue was embedded in paraffin and sectioned at 5 μm. Lung tissue sections were stained with hematoxylin and eosin stain (H&E) for general morphology and identification of eosinophilia and with Alcian blue/periodic acid–Schiff (AB-PAS) for the identification of mucous and goblet cells in the epithelium. The preparation of paraffin block and staining of tissue section were prepared by Logone Bio., Inc. (Seoul, Korea). The stained tissue was observed under microscope equipped with slide scanner SNC400F (Leica Biosystems, Buffalo Grove, IL, USA) and representative images of 3 mice/group were selected. The number of eosinophils was calculated by a blind expert examination.

### 2.5. RNA Preparation and Quantitative Reverse Transcriptase-Polymerase Chain Reaction (qRT-PCR)

To further investigate the mechanism related to the reduction in eosinophilia in the PGT treatment, we examined the gene expression levels, transcription factors, and a surface marker in lung tissues. After 24 h from last challenge (on day 21), the mouse lung was collected in RNA*later^®^* (Ambion, Inc., Austin, TX, USA) solution (500 µL/200 mg lung tissue) and stored −70 °C until analysis. RNA was extracted from lung tissue using easy-spin™ (DNA free) Total RNA Extraction Kit (iNtRON Biotechnology, Seoul, Korea). Complementary DNA (cDNA) was synthesized using a High-Capacity cDNA Reverse Transcription Kit (Thermo Fisher Scientific, San Jose, CA, USA). Synthesized cDNA was used to perform quantitative PCR with Power SYBR^®^ Green Master Mix (Thermo Fisher Scientific, San Jose, CA, USA) on the Applied Biosystems™ QuantStudio™ 6 Flex Real-Time PCR System (Applied Biosystems, Foster City, CA, USA). Primer sequences are shown in Supplementary Table S1.

### 2.6. Determination of Serum IgE

After 24 h from last challenge (on day 21), blood was collected by cardiac puncture immediately after mice were anaesthetised by isoflurane. The blood was coagulated for 30 min at room temperature and centrifuged subsequently at 13,000× *g* for 5 min. Collected serum was stored at −80 °C until further analysis. LEGEND MAX™ Mouse OVA Specific IgE ELISA Kit (Biolegend, San Diego, CA, USA) was used to quantify the IgE levels in serum. The assay was performed according to the manufacturer’s instructions.

### 2.7. Flow Cytometry Analysis

Murine lungs were chopped and incubated in RPMI1640 media with 1 mg/mL type IV collagenase (Worthington BioChem, Lakewood, NJ, USA) for at least 1 h 30 min at 37 °C. Red blood cells (RBC) were lysed using RBC lysis buffer (Biolegend, San Diego, CA, USA). Lung single cells were stained with following fluorochrome-conjugated antibodies. For surface staining, the following antibodies were used. Anti-CD45 (30-F11), Anti-CD3e (145-2C11), anti-CD11c (HL3), anti-CD11b (M1/70), anti-CD19 (ID3), anti-CD49b (DX5), anti-FcεRIα (MAR-1), anti-CD90,2 (30-H12), anti-F4/80 (BM8) and anti-Ly6G (1A8), purchased from Biolegend. Anti-SiglecF (E50-2440) was purchased from BD Bioscience (San Diego, CA, USA). For intracellular staining, the following antibodies were used: anti-IL5 (TRFK5) and anti-IL17A (TC11-18H10.1), purchased from Biolegend (San Diego, CA, USA). Anti-IL13 (eBio13A) was purchased from Thermo Fisher Scientific. For intracellular staining, a Fixation/Permeabilization Solution Kit with BD GolgiPlug (BD Biosciences, San Diego, CA, USA) was used following the manufacturer’s protocol. Flow cytometry was carried out using LSRFortessa™ X-20 (BD biosciences, San Jose, CA, USA) and analyzed by FlowJo (V10.2) software (BD biosciences, San Jose, CA, USA). The gating strategy for immune cell population was represented in Supplementary Figure S1.

### 2.8. In Vitro Experiment with Lung Epithelial Cells and Allergen

Murine lung epithelial cells (MLE-12 ATCC^®^ CRL-2110™) were obtained from the American Type Culture Collection (ATCC, Manasaas, VA, USA) and cultured in DMEM/F-12 medium with the following supplements: 5 µg/mL insulin, 10 µg/mL apo-transferrin, 30nM sodium selenite, 10 nM hydrocortisone, 10 nM β-estradiol, 2 mM l-glutamine, 10mM HEPES, and 10% fetal bovine serum. Papain (Sigma-Aldrich, St Louis, MO, USA) was reconstituted with sterile PBS to 1 mg/mL and stored at −20 °C. In this study, cells were treated with 0.25 mg/mL of papain for stimulation. After 9 h, the cells were collected and lysed. mRNA was extracted using easy-spin™ (DNA free) Total RNA Extraction Kit (iNtRON Biotechnology, Seongnam, Korea) and complementary DNA (cDNA) was synthesized subsequently using Topscript™ RT DryMIX (enzynomics, Daejeon, Korea).

### 2.9. Statistical Analysis

Differences between each group were compared using unpaired Student’s *t*-test, as appropriate, or one-way analysis of variance (version 8.0, GraphPad software, San Diego, CA, USA). In all experiments, the sample size of each group was *n* = 8, 9, and 8 in saline-challenged PBS treatment group (naïve, No-OVA-PBS), OVA-challenged PBS treatment group (vehicle control, OVA-PBS), and OVA-challenged PGT mixture treatment group (OVA-PM), respectively. All values are expressed as means ± standard error of the mean (SEM). Statistical difference was accepted at *p* < 0.05.

## 3. Results

### 3.1. Non-Target Metabolites Profiling of PGT

A total of 793 compounds were identified from the PGT extract, as shown in Supplementary Table S2. The non-targeted metabolites analysis with UPLC and high-resolution mass spectrometry showed a qualitative global profiling showed different classes of herbal components including peptides, sugars, organic acids and a variety of derivatives of alkaloids, saponins, flavonoids, phenols, and plant glycosides.

### 3.2. PGT Treatment Suppressed Airway Inflammation, Mucous Production and Goblet Cell Hyperplasia in OVA-Induced Allergic Asthma Model

**Figure 2:**
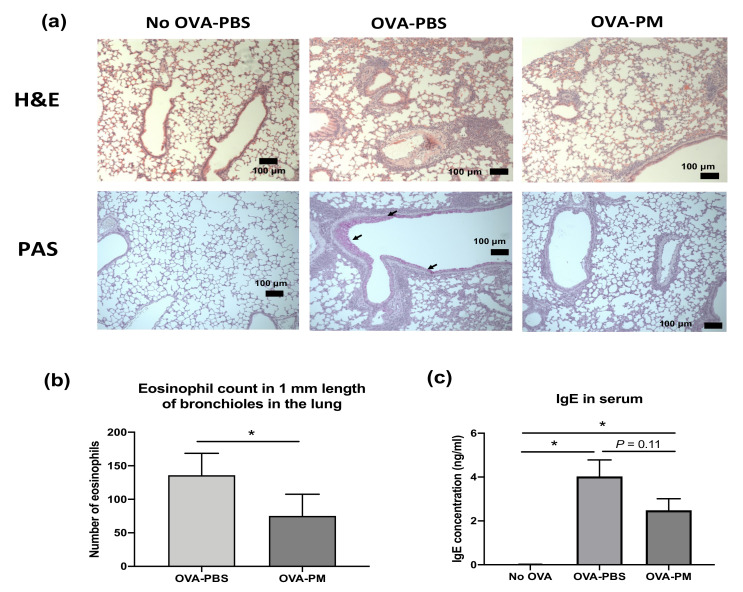
Experimental scheme and effect of PGT on lung histopathology, eosinophil counts in bronchioles in the lung, and lgE levels in serum of mice. Experimental scheme. (**a**) Representative scans of hematoxylin and eosin (H&E) and periodic acid–Schiff (PAS) stained formalin-fixed paraffin-embedded sections of right middle lobe of lungs. (**b**) Number of eosinophils counted in 1 mm length of bronchioles in the lung from H&E stained formalin-fixed paraffin-embedded sections. (**c**) Immunoglobulin E (IgE) levels in serum of mice. Capped bars illustrate the maximum data points within each treatment group. No OVA-PBS group, naïve mouse with PBS oral gavage; OVA-PBS group, OVA-sensitized with PBS oral gavage; OVA-PM group, OVA-sensitized with PGT oral gavage. Data are represented as means ± standard error of the mean (SEM). * *p* < 0.05.

During the study, there was no morbidity or mortality observed in any treatment groups. As expected, in OVA-challenged mice, eosinophil infiltration in the perivascular and peribronchial areas (H&E staining) and overproduction of mucous and goblet cell hyperplasia (AB-PAS staining) were observed (Figure 2a). In PGT-treated mice, the eosinophil infiltration was markedly reduced, and the overproduction of mucous and goblet cell hyperplasia was suppressed (Figure 2a). While no infiltrated eosinophil was observed in the bronchioles of PBS-challenged mice, increased eosinophil counts were found in OVA-challenged mice (Figure 2b). PGT-treated mice showed a significant reduction in the number of eosinophils in comparison to PBS-treated mice. The level of serum immunoglobulin E (IgE) in PGT treatment mice was reduced in comparison to OVA-challenged PBS treatment mice (Figure 2c).

### 3.3. PGT Treatment Alleviated Eosinophilia Infiltration in Lung

**Figure 3:**
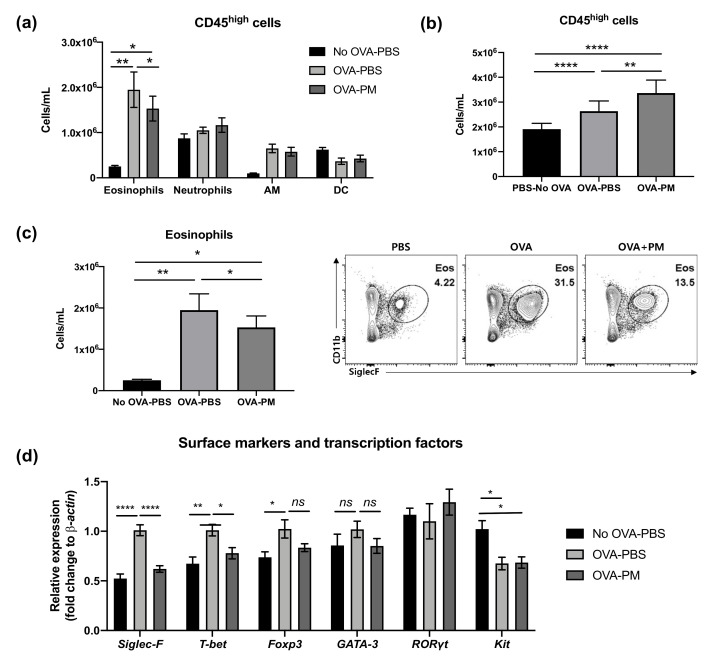
Effect of PGT on lung cell CD45+ counts and gene expression of surface markers and transcription factors. Lung cell counts of mice challenged with OVA, and treated with PGT, with samples collected 24 h after final challenge. (**a**) CD45+ cell subtype counts of each treatment group. (**b**) Total CD45+ cell counts. (**c**) Total eosinophils counts and representative flow cytometry analysis plots of treatment group. (**d**) Relative gene expression levels of cell surface markers and transcription factors in the lung. β-actin was used as a housekeeping gene. No OVA-PBS group, naïve mouse with PBS oral gavage; OVA-PBS group, OVA-sensitization and PBS oral gavage; OVA-PM group, OVA-sensitization and PGT oral gavage. Data are represented as means ± standard error of the mean (SEM). AM, alveolar macrophages; DC, dendritic cells. * *p* < 0.05, ** *p* < 0.01, **** *p* < 0.0001.

Flow cytometry analysis was performed to examine CD45^high^ myeloid cell populations in the lung, which can be used as key markers of pathogenesis of allergic asthmatic inflammation. The populations of eosinophils and neutrophils increased in both OVA-challenged groups (Figure 3a). In contrast, alveolar macrophages (AM) were significantly reduced by OVA challenge. Interestingly, both PBS- and OVA-challenged mice showed no difference in neutrophil populations. All OVA-challenged groups had significantly higher numbers of CD45+ cells than PBS-challenged group (Figure 3b). However, the PGT treatment group (OVA-PM) showed reduced eosinophils in the lung compared to OVA-challenged PBS treatment mice (Figure 3c). This reduction in eosinophils in PGT treatment mice was further confirmed by using RT-PCR analysis (Figure 3d). In OVA-challenged mice, the levels of *Siglec-F*, *T-bet* and *Foxp3* mRNA expression were enhanced in comparison with the PBS-challenged mice; no difference in the levels of *GATA-3* and *RORγt* was detected. In PGT treated mice, we observed a significant reduction in the levels of *T-bet* and *Foxp3,* transcription factors which induce type 1 helper cell (Th1) and T regulatory (Treg) cells, respectively. Notably, the expression of *Siglec-F*, the prominent biomarker of eosinophils, significantly abrogated on a level comparable to that in PBS-challenged group. Both OVA-challenged groups showed a trend to reduce the gene expression of *kit*, the mast cell activation marker.

### 3.4. PGT Treatment Reduced Production of IL-5 and IL-13 in Lung

**Figure 4:**
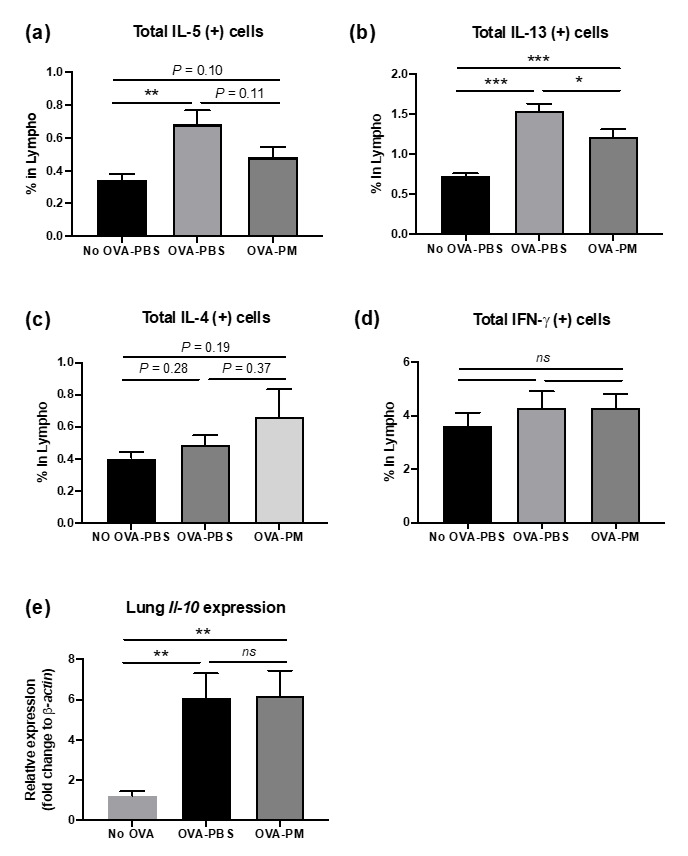
Impact on PGT on lung inflammatory mediators. Percentages of cytokine producing lymphocytes were determined using Fluorescence-activated Cell Sorting (FACS) analysis in lungs of mice challenged with OVA, and treated with PGT, with samples collected 24 h after final challenge. (**a**) % total IL-5 producing lymphocytes. (**b**) % total IL-13 producing lymphocytes. (**c**) % total IL-4 producing lymphocytes. (**d**) % total IFN-γ producing lymphocytes. (**e**) Relative expression level of *Il-10* in lungs determined through RT-qPCR using β-actin as a housekeeping gene. No OVA-PBS group, naïve mouse with PBS oral gavage; OVA-PBS group, OVA-sensitization and PBS oral gavage; OVA-PM group, OVA-sensitization and PGT oral gavage. Data are represented as means ± standard error of the mean (SEM) * *p* < 0.05, ** *p* < 0.01, *** *p* < 0.001.

Flow cytometry analysis of lung lymphocytes was performed to further investigate the mechanisms underlying population change in lung eosinophils made by OVA-induced allergic airway inflammation. We also aimed to detect if the cytokine expression patterns in the lung occurred in correspondence with what we observed in Figure 3b,c. Flow cytometry results displayed increased total amounts of IL-13 and IL-5 in OVA-challenged mice in comparison to PBS-challenged group (Figure 4b). PGT treated mice showed a significant reduction in total IL-5 and IL-13 in lung lymphocytes. Interestingly, despite being a Th2 cytokine, the total amount of IL-4 in lung lymphocytes remained unchanged after the OVA challenge (Figure 4c). We examined the total amount of inflammatory cytokines in lung lymphocytes. Both PBS- and OVA-challenged mice displayed no difference in total amount of Interferon (IFN)-γ, a proinflammatory cytokine, which is produced by Th1 CD4+ cells, in lung lymphocytes (Figure 4d). Next, using RT-PCR, we investigated mRNA relative expression of *Il-10*, a prominent anti-inflammatory cytokine produced by Treg cells. The expression of *Il-10* increased in both OVA-challenged groups, however, no difference was observed between two groups (Figure 4e).

### 3.5. PGT Treatment Significantly Reduced IL-5-Producing Innate Lymphoid Cells

**Figure 5:**
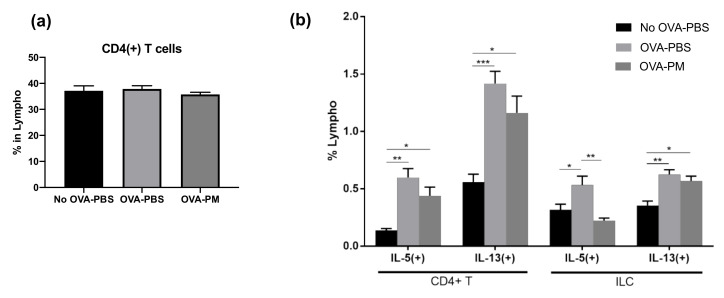
Impact of PGT on populations of CD4+ T cells and innate lymphoid cells (ILCs). Population of cells in lungs of mice challenged with OVA, and treated with PGT, with samples collected 24 h after final challenge. Percentages of cytokine producing lymphocytes were determined using Fluorescence-activated Cell Sorting (FACS). (**a**) % of total CD4+ T cells, (**b**) IL-5(+) and IL-13(+) subtypes of CD4+ T cells (left) and ILCs (right). No OVA-PBS group, naïve mouse with PBS oral gavage; OVA-PBS group, OVA-sensitization and PBS oral gavage; OVA-PM group, OVA-sensitization and PGT oral gavage. Data are represented as means ± standard error of the mean (SEM) * *p* < 0.05, ** *p* < 0.01, *** *p* < 0.001.

We confirmed that the OVA-induced allergic asthma is mainly driven by the Th2 response, following the production of IL-5 and IL-13, but not IL-4, and that PGT treatment greatly reduced such induction. We then investigated which immune cell populations were targeted by PGT. Interestingly, we detected no difference in the composition of CD4+ T cell populations among PBS-challenged groups and OVA-challenged groups (Figure 5a). Still, although not statistically significant, there was a trend which suggested a reduced population of IL-5 and IL-13 producing CD4+ T cells in the PGT treatment group (Figure 5b). Next, we sought for different groups of cells in addition to T lymphocytes that can produce IL-5 and IL-13. We thus investigated how OVA-induced allergic asthma changed innate lymphoid cells (ILCs) in the lungs (Figure 5b). Interestingly, populations of IL-5 producing ILCs were significantly reduced in the PGT treatment group compared with the OVA-challenged control groups (Figure 5b). Overall, the results of immune profile analysis showed that PGT treatment reduced the populations of IL-5 and IL-13 producing immune cells, such as CD4+ T cells and ILCs, as well as the total amount of IL-5 and IL-13 producing cells in OVA-induced allergic asthma.

### 3.6. PGT Treatment Suppressed Production of IL-33, But not TSLP, in Murine Lung Epithelial Cells after Papain Stimulation

**Figure 6:**
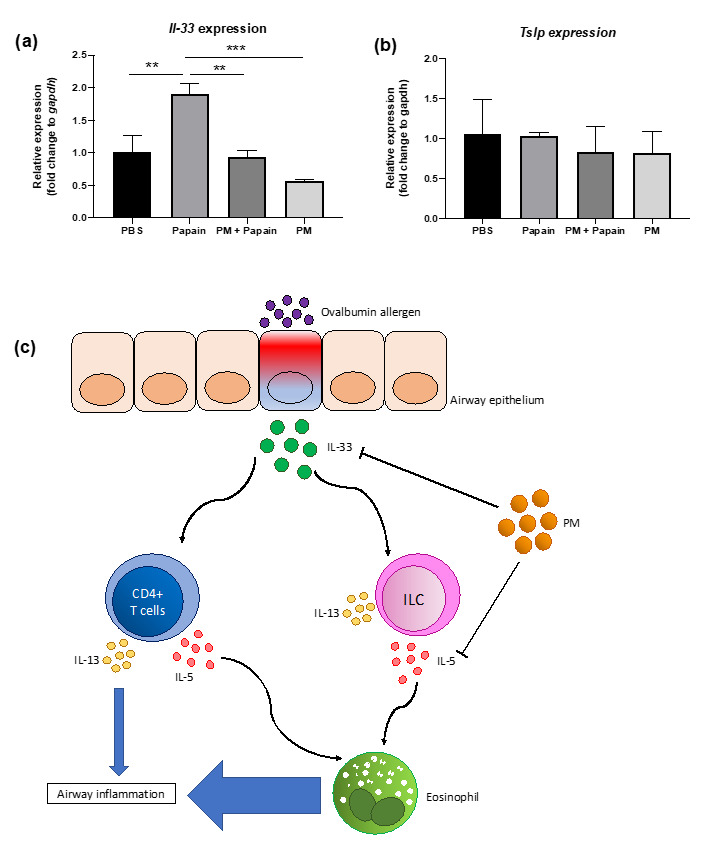
Effects of PGT on IL-33 (**a**) and thymic stromal lymphopoietin (TSLP) (**b**) expression in epithelial cells in vitro, and schematic diagram of its plausible mechanism (**c**). MLE-12 cells were grown to confluence and stimulated with PBS or papain (0.25 mg/mL), followed by either PBS or PGT (1 mg/mL) treatment. Cells were harvested after 9 h. *Gapdh* gene was used to normalize the expression levels between groups. PBS group, PBS and PBS treated; papain group, papain and PBS treated; PM+papain group, papain and PGT treated; PM group, PBS and PGT treated. Data are represented with means ± standard error of the mean (SEM). ** *p* < 0.01, *** *p* < 0.001.

A significant reduction in IL-5(+) ILC cell populations, shown in Figure 5, led us to conduct a further investigation of the mechanism through which PGT induced such a change. We set up an in vitro experiment using MLE-12 cells, using papain as stimulant. Notably, PGT treatment significantly reduced IL-33 mRNA expression, to an even greater degree than the control treatment (Figure 6a) in the absence of papain stimulation. Under the papain stimulation, addition of PGT significantly reduced the IL-33 mRNA expression. In contrast, thymic stromal lymphopoietin (TSLP), another cytokine produced by lung epithelial tissue under allergen stimulation (Figure 6b), showing that the PGT specifically targets the production of IL-33. A possible mechanism of PGT on alleviation of airway inflammation, by supressing asthmatic eosinophil recruitment and infiltration in the lung, is represented in Figure 6c.

## 4. Discussion

In this study, we demonstrated the effect of PGT on significantly reducing the disease pathogenesis in OVA-induced allergic asthma model in mice. As an exploratory experiment, we also performed the global non-target metabolites profiling using UPLC-MS analysis to provide an overview of the constituents in complex herbal remedies. Although a total of 793 compounds were identified from the data of three PGT samples, it is difficult to conclude which molecules are active components of PGT, exerting anti-asthmatic functions. However, qualitative characterization of the herbal mixture could be applied for comparative analysis of different plant compounds and quantitative assessment of bioactive compounds in further studies.

Ovalbumin is a well-characterized and widely-used allergen to induce acute allergic asthma in mice. OVA induction increased pulmonary inflammatory infiltrates, despite the reduction in alveolar macrophages. However, a striking effect of PGT on the significant reduction in lung eosinophilia was confirmed through lung histopathology and flow cytometry. This suggests that the disease was induced in a strongly eosinophil-dependent manner. Consistent with this finding, a significant reduction in mRNA expression levels of *siglec-F*, a surface marker of eosinophil which is used to regulate levels of eosinophils, was also observed.

Our flow cytometry results revealed that the effects of PGT on reducing OVA-induced eosinophilia are closely related with regulating the levels of IL-5 and IL-13 in the lung. Previous studies have shown that various types of helper T cells contribute to the pathogenesis of allergic asthma [38,39,40]. Multiple mechanisms have been proposed to provide insight into our understanding of the development of the disease and resulting phenotypes, which eventually, suggest potential therapeutic targets. Th2 asthma hypothesis, which is the most prominently used to explain the disease development, is based on dysregulation of the Th1/Th2 balance [41]. It states that IgE and eosinophils are regulated by Th2 cytokines and play a major part in disease pathogenesis. Th2 cells activated by allergen produce Th2 cytokines, mainly IL-4, IL-5 and IL-13, which leads to eosinophilic lung inflammation, goblet cell hyperplasia, and mucous production. The recruitment of eosinophils in the lung and the degree of eosinophilia are closely related to AHR.

IL-5 is a cytokine which has a high specificity for eosinophil production, differentiation and activation. The prime source of IL-5 is Th2 lymphocytes of adaptive immunity as the Th2 hypothesis states, but other sources of IL-5 are also present. ILC2 have been reported to significantly contribute to IL-5 and IL-13 production, thus activating and recruiting eosinophils [42]. ILCs may act as the innate counterparts of Th1, Th2, and Th17 cells, yet they lack antigen receptors [43]. While allergens presented by dendritic cells induce the differentiation of CD4+ naïve T-cells into Th2 cells, cytokines secreted by lung epithelial cells, such as IL-33, IL-25, and thymic stromal lymphopoietin (TSLP) directly activate ILCs [43,44]. Our results display that both T cells and ILCs seem to contribute to the production of IL-5 and IL-13 when OVA in alum induced asthma, suggesting both immune response pathways of Th2 cells and ILCs can be therapeutic targets for preventing or treating asthma.

Among the Th2 cell-related cytokines, IL-4 plays a crucial role in the differentiation of naïve T cells to Th2 cells. PGT reduced the total levels of IL-5 and IL-13 in the lung, but not IL-4. Furthermore, the mRNA level of GATA-3, the signature transcription factor of Th2 cells, was not affected by PGT. These results suggest that the main target of PGT’s immune-modulation was not dependent on the classical Th2 response that is activated by the introduction of allergens by DCs to naïve CD4+ T cells. Although a significant reduction in *T-bet* was observed in the PGT treatment group, it is less likely to be due to Th1/Th2 dysregulation, as the levels of *IFN-γ* remained unchanged. Moreover, no significant difference in the levels of *IL-10* between to OVA-challenged groups suggests that T regulatory (Treg) cells were not the main target of the effect of PGT. Therefore, the reduced levels of IL-5 and IL-13 producing cells could be attributed to the modulatory effect of PGT on an ILC pathway.

Indistinguishable population differences of CD4+ T cells among the three mice groups and modest decreases in the populations of both IL-5+ -and IL-13+ CD4+ T cells in PGT-treated group in comparison to PBS-treated group provide more concrete evidence for the modulating effect of PGT on the ILC pathway. Our results provide insufficient evidence to prove the efficacy of PGT on the disease progress only by modulating the adaptive type-2 immune response. Instead, efficacy of PGT was reflected in a significant reduction in the population of IL-5+ ILCs, which explains the reduced levels of total IL-5 in the lungs of PGT-treated mice, and even further, reduced disease severity. There is growing evidence that ILCs can act upstream of the immune pathway involving adaptive type 2-immune response [45]. ILC2-deficient mice lacked the ability to induce the adaptive type 2-immune response in response to the papain allergen [42]. While large amounts of IL-13 and IL-5 were produced by ILC2 under papain stimulation, change in the level of IL-4 was negligible; the mechanism of papain-induced Th2 responses was independent of IL-4. IL-13 produced by ILC acted as a main promoter of Th2 cell differentiation in the draining lymph nodes [42]. Coincidingly, our results suggest a role of PGT on production of IL-5 and IL-13 by ILC under OVA stimulation, which then interferes with induction of the Th2 responses and eosinophils, ultimately reducing the disease severity.

We hypothesized that orally administered PGT might directly affect the lungs through the bloodstream, and as a result, the levels of alarmin cytokines secretion by lung epithelial cells under allergen stimulation will be altered. The alarmin cytokines, such as IL-33 and TSLP, activate and expand the ILC2, which then will produce IL-5 and IL-13 to induce eosinophil activation and other phenotypic changes. Significant reduction in *il-33* expression in PGT-treated MLE-12 cells under papain stimulation, suggest a role of PGT in regulating IL-33 production and in earlier stages of allergic asthma development. IL-33 is an important cytokine for the regulation of both the innate and adaptive immunity. It stimulates Th2 differentiation of naïve CD4^+^T lymphocytes in an IL-4 independent manner, enhancing IL-5 and IL-13 production [46]. It also regulates development and the function of ILC2, as IL-13 production in chronic rhinosinusitis with nasal polyps is largely dependent on epithelial cell-derived IL-33 and IL-33-responsive ILC [47]. Our results suggest that PGT may directly act on lung epithelial cells and down-regulate their IL-33 production, leading to downregulation of the T_H_ response and ILC response, and overall disease development.

Taken together, our results showed the effectiveness of PGT as herbal remedies to control the development of OVA-induced asthma. We showed the reducing effects of PGT on type 2 immunity and lung eosinophil infiltration in OVA-induced allergic asthma. Additionally, we successfully identified that PGT lowers ILCs and CD4+ populations which produce IL-5 and IL-13 in an IL-4 independent manner, which results in lowering eosinophil recruitment in the lungs, leading to decreasing the overall disease severity. We used OVA to induce a strong Th2 response, our results suggest that PGT may be act on upstream of Th2 response by inducing IL-33 production and ILCs.

## 5. Conclusions

In conclusion, the results suggest the possibility of using PGT as an alternative treatment to asthma. It is the first study to evaluate the mechanism of PGT in the reduction in airway infection. In the future, the bioactive compounds in the PGT mixture will be identified, and the protective effects of PGT will be carried out in non Th2-type asthma models.

## Figures and Tables

**Table 1:** Composition of Pyunkang-tang (PGT) herbal remedy.

Common Name	Genus	Family	Deposit No.	Dry Weight (g)
Lonicerae Flos *(flowers)*	*Lonicerae japonica* Thunberg	Caprofoliaceae	KHUOPS 2013-75	4
Liriopis Tuber *(underground stem/roots)*	*Liriope platyphylla* Wang et Tang	Liliaceae	KHUOPS 2013-76	4
Adenophorae Radix *(roots)*	*Adenophora triphilla* var. *japonoica* Hara	Campanulaceae	KHUOPS 2013-77	10
Xanthii Fructus *(fruits)*	*Xantium strumarinum* Linne	Compositae	KHUOPS 2013-78	10
Selaginellae Herba *(leaves)*	*Selaginella tamariscina* Spring	Selaginellaceae	KHUOPS 2013-79	10
Rehmanniae Radix Preparata *(roots)*	*Rehmannia glutinosa* Liboschitz var. *purpurea* Making	Scrophulariaceae	KHUOPS 2013-80	2

## Supplementary Materials

The following are available online at https://www.mdpi.com/2076-3921/10/1/6/s1, Figure S1: Gating strategy for flow cytometry, Table S1: PCR primers used in this study, Table S2: Full chemical components of PGT analyzed by non-targeted UPLC/MS system.
