# Supplementary material for: Protective Effects of Korean Herbal Remedy against Airway Inflammation in an Allergic Asthma by Suppressing Eosinophil Recruitment and Infiltration in Lung
Source: Antioxidants (Basel). 2020 Dec 23;10(1):6. doi: 10.3390/antiox10010006 (PMC7822450; doi:10.3390/antiox10010006)
Supplement: Supplementary file 1 [file antioxidants-10-00006-s001.zip › antioxidants-1003427-supplementary-author proofed.docx]

**Supplementary materials**

**Supplementary Table S1.** PCR primers used in this study

| **Target** | **Forward** | **Reverse** | | **Accession number** |
| --- | --- | --- | --- | --- |
| β-actin | AGA GGG AAA TCG TGC GTG AC | CAA TAG TGA TGA CCT GGC CGT |  | X03672 |
| GAPDH | AGG TCG GTG TGA ACG GAT TTG | TGT AGA CCA TGT AGT TGA GGT CA |  | X02231 |
| Siglec-F | CTG GCT ACG GAC GGT TAT TC | GGA ATT GGG GTA CTG GAC TTG | | AK146203.1 |
| T-bet | TTC CCA TTC CTG TCC TTC AC | CCA CAT CCA CAA ACA TCC TG | | AF093099.1 |
| GATA3 | GGA AAC TCC GTC AGG GCT A | AGA GAT CCG TGC AGC AGA G | | Z33620.1 |
| FoxP3 | CCT GCC TTG GTA CAT TCG TG | TGT TGT GGG TGA GTG CTT TG | | AB673115.1 |
| RORγT | TGA GGC CAT TCA GTA TGT GG | CTT CCA TTG CTC CTG CTT TC | | U43088.1 |
| IL-33 | TGA GAC TCC GTT CTG GCC T | CTC TTC ATG CTT GGT ACC CG | | AY905582.1 |
| IL-10 | ATT TGA ATT CCC TGG GTG AGA AG | CAC AGG GGA GAA ATC GAT GAC | | AK152344.1 |

**Supplementary Figure S1.** Gating strategy for flow cytometry
