# Supplementary material for: Protective Effects of Korean Herbal Remedy against Airway Inflammation in an Allergic Asthma by Suppressing Eosinophil Recruitment and Infiltration in Lung
Source: Antioxidants (Basel). 2020 Dec 23;10(1):6. doi: 10.3390/antiox10010006 (PMC7822450; doi:10.3390/antiox10010006)
Supplement: Supplementary file 1 [file antioxidants-10-00006-s001.zip › antioxidants-1003427-supplementary table S2.pdf]

Primary ID	Neutral mass (Da)	m/z	Retention time (min)	Accepted Description	Accepted ID
0.08_158.9681m/z		158.9681	0.08	Allyl methyl selenide	CSID10338214
0.45_315.8585m/z		315.8585	0.45	3,5-Dibromo-4-hydroxybenzamide	CSID2282682
0.45_496.9419m/z		496.9419	0.45	aerophobin 1	CSID8226207
0.45_440.9215m/z		440.9215	0.45	Calcium dobesilate	CSID27837
0.45_306.5211m/z		306.5211	0.45	Chikusetsusaponin III	CSID29273184
0.46_390.8956m/z		390.8956	0.46	Triclosan sulfate	CSID30778644
0.47_323.1870m/z		323.187	0.47	Tilmicosin phosphate	CSID4445657
0.48_258.8963m/z		258.8963	0.48	Methyl 2,3-dibromo-2-methylpropanoate	CSID465481
0.48_266.9374m/z		266.9374	0.48	Rhodanine	CSID1013337
0.49_159.0248m/z		159.0248	0.49	2,4-Difluorobenzoic acid	CSID66716
0.49_196.0032m/z		196.0032	0.49	Phenylsulfamic acid	CSID381241
0.50_296.9564m/z		296.9564	0.5	1-O-Arsonopentofuranose	CSID35032430
0.50_311.1825m/z		311.1825	0.5	6-Hydroxypentadecanedioic acid	CSID35013393
0.50_201.1295m/z		201.1295	0.5	a-yl decanoate	CSID4444745
0.50_159.0926m/z		159.0926	0.5	bamipine hydrochloride	CSID108372
0.50_223.9985m/z		223.9985	0.5	carzenide	CSID8410
0.50_280.1626m/z		280.1626	0.5	Chivosazole C	CSID10218297
0.50_429.2336m/z		429.2336	0.5	Diammonium Glycyrrhizinate	CSID571005
0.50_452.9233m/z		452.9233	0.5	Dichlone	CSID8039
0.50_279.1572m/z		279.1572	0.5	Dronedarone	CSID180996
0.50_261.0441m/z		261.0441	0.5	Fluorouracil	CSID3268
0.50_654.8783m/z		654.8783	0.5	Iodohippurate sodium	CSID8294
0.50_192.1202m/z		192.1202	0.5	Pyrvinium	CSID21125
0.50_213.1286m/z		213.1286	0.5	Synaptolepis factor K1	CSID4444751
0.50_123.0737m/z		123.0737	0.5	Veliparib	CSID10134775
0.51_124.0805m/z		124.0805	0.51	3-Hydroxydodecanedioic acid	CSID13628081
0.51_366.2068m/z		366.2068	0.51	6-O-DesmethylDonepezil	CSID21627304
0.51_396.2179m/z		396.2179	0.51	benzyl cetraxate	CSID21864808

0.51_154.0935m/z	154.0935	0.51 fenazaquin	CSID77874
0.51_164.1074m/z	164.1074	0.51 hydroquinidine	CSID391215
0.51_556.2785m/z	556.2785	0.51 Leu-enkephalin	CSID406229
0.51_269.0624m/z	269.0624	0.51 L-Homocystine	CSID388664
0.51_527.3428m/z	527.3428	0.51 melperone	CSID14646
0.51_146.0882m/z	146.0882	0.51 Meptin	CSID4747
0.51_572.2951m/z	572.2951	0.51 Moxilubant maleate	CSID8135368
0.51_585.2892m/z	585.2892	0.51 Ouabain	CSID388599
0.51_394.2147m/z	394.2147	0.51 Tamoxifen	CSID2015313
0.51_147.1192m/z	147.1192	0.51 Triethyl borate	CSID8659
0.52_729.2991m/z	729.2991	0.52 (1R)-2-Phenylcyclopropanamine sulfate (2:1)	CSID28290625
0.52_806.2963m/z	806.2963	0.52 Golvatinib tartrate	CSID30790729
0.52_833.2931m/z	833.2931	0.52 halopemide	CSID58938
0.52_713.3157m/z	713.3157	0.52 Hycanthone	CSID3508
0.52_757.3045m/z	757.3045	0.52 Isouvaretin	CSID133675
0.52_459.2515m/z	459.2515	0.52 lifarizine	CSID64440
0.52_991.3358m/z	991.3358	0.52 Maltohexaose	CSID388684
0.52_817.2943m/z	817.2943	0.52 Nodakenin	CSID65952
0.52_927.3123m/z	927.3123	0.52 Pelitrexol	CSID4953371
0.52_291.1681m/z	291.1681	0.52 Terit	CSID35605
0.53_798.3046m/z	798.3046	0.53 2''-Adenylylgentamicin A	CSID30791501
0.53_653.2992m/z	653.2992	0.53 aceprometazine	CSID24249
0.53_131.0943m/z	131.0943	0.53 carisoprodol	CSID2478
0.53_513.2627m/z	513.2627	0.53 DEXAMETHASONE TEBUTATE	CSID16736122
0.53_207.0561m/z	207.0561	0.53 Isoflurophate	CSID5723
0.53_499.2448m/z	499.2448	0.53 lofentanil oxalate	CSID58946
0.53_491.2724m/z	491.2724	0.53 Ziziphin	CSID30791203
0.54_347.1345m/z	347.1345	0.54 Deutzioside	CSID391588
0.54_236.9150m/z	236.915	0.54 dimexano	CSID14387
0.55_221.0283m/z	221.0283	0.55 (E)-Nitrofurazone	CSID4566720
0.55_143.0375m/z	143.0375	0.55 4-Chloro-1,3-benzenediamine	CSID19941

0.55_365.1207m/z	365.1207	0.55 Abietin	CSID4444067
0.55_177.0313m/z	177.0313	0.55 Butadiynyl phenyl ketone	CSID515451
0.55_223.0292m/z	223.0292	0.55 camalexin	CSID552646
0.55_214.0152m/z	214.0152	0.55 Carmustine	CSID2480
0.55_1013.1093m/z	1013.109	0.55 cefditoren	CSID8046534
0.55_333.9784m/z	333.9784	0.55 Diclofenac potassium	CSID59753
0.55_541.0243m/z	541.0243	0.55 dithiazanine iodide	CSID4642986
0.55_311.0388m/z	311.0388	0.55 Furamizole	CSID4527168
0.55_933.4574m/z	933.4574	0.55 Indolapril hydrochloride	CSID8632692
0.55_304.8466m/z	304.8466	0.55 Samarium	CSID22391
0.56_381.0945m/z	381.0945	0.56 Chryso-obtusin	CSID136879
0.56_341.0379m/z	341.0379	0.56 Thiourocanic acid	CSID4444356
0.57_219.0358m/z	219.0358	0.57 1-Chloro-1-phenylsilolane	CSID9416070
0.57_955.3555m/z	955.3555	0.57 Batimastat	CSID4515033
0.57_242.9350m/z	242.935	0.57 dichlorvos	CSID2931
0.57_293.0784m/z	293.0784	0.57 pinostrobin	CSID65961
0.57_164.0369m/z	164.0369	0.57 Tiopronin	CSID5283
0.58_406.9754m/z	406.9754	0.58 ethion	CSID3171
0.58_286.9644m/z	286.9644	0.58 pralidoxime iodide	CSID13233097
0.59_202.9984m/z	202.9984	0.59 7-chloroquinazolin-4-ol	CSID208976
0.59_351.0799m/z	351.0799	0.59 Milneb	CSID83240
0.59_203.0614m/z	203.0614	0.59 protionamide	CSID579891
0.61_252.9822m/z	252.9822	0.61 6,7-Dichloro-2-dibenzofuranol	CSID10128421
0.61_162.0582m/z	162.0582	0.61 entadamide A	CSID4943641
0.61_132.9932m/z	132.9932	0.61 Methyl mesylate	CSID4013
0.62_471.0646m/z	471.0646	0.62 Calcium Gluconate Monohydrate	CSID24590783
0.62_183.1197m/z	183.1197	0.62 Heptyl thiol	CSID79206
0.63_521.0464m/z	521.0464	0.63 6-O-Sulfo-alpha-D-galactopyranose	CSID388504
0.63_425.0081m/z	425.0081	0.63 Triafur	CSID55149
0.64_297.0070m/z	297.007	0.64 Fenclofenac	CSID58860
0.64_409.0245m/z	409.0245	0.64 guaiacol sulfate	CSID21078
0.65_288.1194m/z	288.1194	0.65 Clavamycin E	CSID30791679

0.67_326.0356m/z		326.0356	0.67	Gerrardine	CSID391000
0.67_477.0114m/z		477.0114	0.67	primisulfuron [ANSI]	CSID82871
0.67_270.9887m/z		270.9887	0.67	Vanillic acid 4-sulfate	CSID339308
0.71_487.0399m/z		487.0399	0.71	ethoate-methyl	CSID7996
0.71_299.0096m/z		299.0096	0.71	N,N,1,2,3-Pentafluoro-3-diaziridinamine	CSID465244
0.72_863.3842m/z		863.3842	0.72	ethaverine hydrochloride	CSID13199
0.72_194.0319m/z		194.0319	0.72	fosfocreatinine	CSID64349
0.72_302.1356m/z		302.1356	0.72	Salcaprozate sodium	CSID139678
0.74_290.1025m/z		290.1025	0.74	Acronycidine	CSID86397
0.77_229.9849m/z		229.9849	0.77	nimidane	CSID58902
0.80_305.1033m/z		305.1033	0.8	heraclenol	CSID290709
0.82_235.1290m/z		235.129	0.82	2,6-Diamino-7-hydroxynonanedioic acid	CSID391749
0.82_207.1082m/z		207.1082	0.82	Adapalene	CSID54244
0.82_326.1401m/z		326.1401	0.82	Amurine	CSID4586084
0.82_329.1023m/z		329.1023	0.82	Betagarin	CSID390742
0.82_297.6796m/z		297.6796	0.82	Bis(5'-guanosyl) tetraphosphate	CSID144813
0.82_193.1066m/z		193.1066	0.82	Bortezomib	CSID343402
0.82_270.1719m/z		270.1719	0.82	Fusidate Sodium	CSID2298842
0.82_253.1285m/z		253.1285	0.82	His-pro	CSID10608298
0.82_123.0553m/z		123.0553	0.82	Isonicotinamide	CSID14346
0.82_262.1408m/z		262.1408	0.82	Macrophylline	CSID4445052
0.82_228.0832m/z		228.0832	0.82	N-Acetyl-D-fucosamine	CSID10128481
0.82_294.1139m/z		294.1139	0.82	Oxaprozin	CSID4453
0.82_209.1378m/z		209.1378	0.82	Propanoylagmatine	CSID388817
0.82_276.0973m/z		276.0973	0.82	triamterene	CSID5345
0.82_147.1188m/z		147.1188	0.82	Triethyl borate	CSID8659
0.82_224.1313m/z		224.1313	0.82	Vaspit	CSID13085382
0.83_517.2864m/z		517.2864	0.83	(+)-thiobinupharidine	CSID390975
0.83_172.1318n	172.1318	195.121	0.83	Acetylagmatine	CSID388732
0.83_320.1850m/z		320.185	0.83	Coronatine	CSID82784

0.83_254.1733m/z	254.1733	0.83	EDC7F4TF12	CSID68969
0.83_348.0887m/z	348.0887	0.83	N-Glycolylneuraminic acid	CSID110352
0.83_383.1939m/z	383.1939	0.83	O-Acetylcypholophine	CSID391156
0.84_359.1962m/z	359.1962	0.84	Risocaine	CSID6906
0.85_339.1689m/z	339.1689	0.85	amquinate	CSID26433
0.85_387.1923m/z	387.1923	0.85	Olodaterol	CSID9679097
0.85_417.2200m/z	417.22	0.85	Zanoterone	CSID8020541
0.87_161.0783m/z	161.0783	0.87	Dimethyl(phenylethynyl)silane	CSID3333176
0.87_478.2223m/z	478.2223	0.87	Lacidipine	CSID4470736
0.88_276.1582m/z	276.1582	0.88	HOMATROPINE	CSID16498795
0.88_248.1595m/z	248.1595	0.88	lys-thr	CSID13177972
0.89_212.1196m/z	212.1196	0.89	mefenorex	CSID20467
0.89_199.0639m/z	199.0639	0.89	Monuron	CSID8470
0.89_258.1463m/z	258.1463	0.89	Stovine	CSID10312
0.97_475.1317m/z	475.1317	0.97	2,4-DIPHENYLTHIAZOLE	CSID536201
0.99_260.2008m/z	260.2008	0.99	Eperisone	CSID3123
0.99_403.1034m/z	403.1034	0.99	Frangulin B	CSID391087
1.00_197.0474m/z	197.0474	1	clominorex	CSID18605
1.00_385.0923m/z	385.0923	1	isoscopoletin	CSID63089
1.00_565.1714m/z	565.1714	1	protoaphin aglucone	CSID26330536
1.02_282.1500m/z	282.15	1.02	Coumarin 106	CSID90670
1.02_203.1061m/z	203.1061	1.02	Tremetone	CSID71024
1.03_423.2235m/z	423.2235	1.03	pinoxaden	CSID182281
1.05_338.1942m/z	338.1942	1.05	Butoctamide semisuccinate	CSID2392
1.05_251.1627m/z	251.1627	1.05	frescolat ML	CSID55982
1.05_499.1442m/z	499.1442	1.05	MTX sodium	CSID571037
1.08_186.1253m/z	186.1253	1.08	6-ketoprostaglandin f1	CSID4444411
1.08_230.0599m/z	230.0599	1.08	alpha-L-Oxathionyl-cytidine	CSID389999
1.08_484.2617m/z	484.2617	1.08	Bekanamycin	CSID388449
1.08_185.1361m/z	185.1361	1.08	Carboprost	CSID4444532
1.09_333.0721m/z	333.0721	1.09	6-Deoxyjacareubin	CSID4444948
1.10_209.1073m/z	209.1073	1.1	spironolactone	CSID5628

1.11_183.1205m/z		183.1205	1.11	Kendall's Compound G	CSID16735652
1.12_279.1473m/z		279.1473	1.12	(8R,9S)-fumigaclavine B	CSID10207493
1.12_153.1091m/z		153.1091	1.12	11-KETOETIOCHOLANOLONE	CSID92021
1.12_328.1812n	328.1812	329.1885	1.12	4-(3,5-Diphenylcyclohexyl)phenol	CSID10128318
1.12_255.1823m/z		255.1823	1.12	Aloperine	CSID10393201
1.12_147.1201m/z		147.1201	1.12	n-Dibutyl sulfide	CSID10536
1.12_168.0767m/z		168.0767	1.12	spinacine	CSID323081
1.14_573.3185m/z		573.3185	1.14	2-Hydroxyestrone	CSID389514
1.14_234.1364m/z		234.1364	1.14	Androsterone glucuronide	CSID102793
1.14_173.0991m/z		173.0991	1.14	Bis(trimethylsilyl) camphorate	CSID29272943
1.14_397.2290m/z		397.229	1.14	Mebicar	CSID109042
1.15_517.2996m/z		517.2996	1.15	3-O-(alpha-L-olivosyl)oleandolide	CSID391741
1.16_379.2186m/z		379.2186	1.16	Cebranopadol	CSID29398942
1.17_528.2957m/z		528.2957	1.17	doramapimod	CSID137746
1.19_308.1856m/z		308.1856	1.19	Buflomedil	CSID2373
1.19_233.1541m/z		233.1541	1.19	Pristimerin	CSID140269
1.21_181.1063m/z		181.1063	1.21	(2S)-2-Carboxy-1,1-dimethylpiperidinium	CSID4444556
1.22_224.0926m/z		224.0926	1.22	DIOXACARB	CSID21901
1.24_475.1321m/z		475.1321	1.24	2,4-DIPHENYLTHIAZOLE	CSID536201
1.24_472.2104m/z		472.2104	1.24	Dabigatran	CSID187412
1.27_292.1326m/z		292.1326	1.27	Naproanilide	CSID36918
1.29_313.0681m/z		313.0681	1.29	Luteoforol	CSID389678
1.29_131.0607m/z		131.0607	1.29	Quinoxaline	CSID21106470
1.29_232.1119m/z		232.1119	1.29	Valerosidatum	CSID8632465
1.32_620.3219m/z		620.3219	1.32	Glycylalanylprolylmethionylphenylalanylvalinamide	CSID35013564
1.36_173.1363m/z		173.1363	1.36	Oxapium	CSID4452
1.38_153.0765m/z		153.0765	1.38	Nopaline	CSID390247
1.39_599.3330m/z		599.333	1.39	Cyclo-(D-Leu-L-Trp)	CSID9360730
1.41_279.1476m/z		279.1476	1.41	(8R,9S)-fumigaclavine B	CSID10207493
1.41_217.1207m/z		217.1207	1.41	1-linolenoyl-sn-glycero-3-phosphate	CSID29368413

1.41_408.2165m/z	408.2165	1.41	ancistrocladine	CSID142055
1.41_193.1197m/z	193.1197	1.41	Diafenthiuron	CSID2298854
1.42_223.1584m/z	223.1584	1.42	Menatetrenone	CSID4445530
1.43_184.1084m/z	184.1084	1.43	6?,9-difluoro-11?-hydroxy progesterone	CSID224469
1.43_202.1279m/z	202.1279	1.43	Telecinobufagin	CSID228185
1.44_440.7524m/z	440.7524	1.44	OCTACHLORODIBENZOFURAN	CSID35020
1.46_240.1498m/z	240.1498	1.46	(-)-2,3-Dehydroemetine	CSID19773
1.46_154.0870m/z	154.087	1.46	Ruxolitinib	CSID25027389
1.47_260.1688m/z	260.1688	1.47	Cucurbitacin F	CSID4444697
1.49_141.0733m/z	141.0733	1.49	3,4-Diethylthiophene	CSID454231
1.49_198.1195m/z	198.1195	1.49	Carfentanil	CSID55986
1.50_169.1413m/z	169.1413	1.5	6-Silaspiro[5.5]undecane	CSID14690870
1.50_359.2031m/z	359.2031	1.5	salirasib	CSID4579849
1.52_467.2569m/z	467.2569	1.52	2-Heptyl-1,3-benzothiazole	CSID30777401
1.52_147.1205m/z	147.1205	1.52	n-Dibutyl sulfide	CSID10536
1.54_226.1380m/z	226.138	1.54	laserpitin	CSID4444857
1.55_289.1687m/z	289.1687	1.55	Desipramine	CSID2888
1.56_301.1417m/z	301.1417	1.56	Dibutyl phthalate	CSID13837319
1.57_248.9828m/z	248.9828	1.57	2,2'-[Sulfanediylbis(methylene)]dithiophene	CSID30776965
1.57_185.1129m/z	185.1129	1.57	Hirsutine	CSID2301518
1.57_183.1200m/z	183.12	1.57	Tetrahydrocortisone	CSID5657
1.58_231.1697m/z	231.1697	1.58	Leu-Val	CSID312585
1.62_141.1098m/z	141.1098	1.62	6-Silaspiro[3.5]nonane	CSID24607727
1.62_305.1883m/z	305.1883	1.62	Retinal 2	CSID4444397
1.63_211.0647m/z	211.0647	1.63	1-(Ethyldisulfanyl)-1-(propylsulfanyl)propane	CSID35013530
1.63_393.2386m/z	393.2386	1.63	Rimexolone	CSID4470902
1.68_244.1422m/z	244.1422	1.68	Alchorneine	CSID188607
1.70_169.1413m/z	169.1413	1.7	6-Silaspiro[5.5]undecane	CSID14690870

1.71_539.9724m/z		539.9724	1.71	AIM	CSID82810
1.71_331.1637m/z		331.1637	1.71	Hydroxycarteolol	CSID111020
1.71_202.1016m/z		202.1016	1.71	Linogliride fumarate	CSID23942945
1.71_168.0880m/z		168.088	1.71	N-cyclopropylammeline	CSID4881949
1.71_163.0494m/z		163.0494	1.71	quindoxin	CSID65059
1.71_229.1112m/z		229.1112	1.71	Tebuthiuron	CSID5190
1.74_297.1060n	297.106	320.1895	1.74	Afegostat tartrate	CSID29324507
1.79_188.0819m/z		188.0819	1.79	Amrinone	CSID3570
1.79_146.0690m/z		146.069	1.79	N,N-Dimethyl-2-pyrimidinamine	CSID455090
1.79_160.0861m/z		160.0861	1.79	N-Isopropyl-2-propanaminium chloride	CSID12616
1.82_257.1985m/z		257.1985	1.82	Sparteine	CSID17340494
1.87_196.1090m/z		196.109	1.87	Megaphone	CSID391211
1.89_561.1174m/z		561.1174	1.89	CEFATRIZINE PROPYLENE GLYCOLATE	CSID4918614
1.89_527.0917m/z		527.0917	1.89	miloxacin	CSID34501
1.91_896.9654m/z		896.9654	1.91	aldesulfone sodium	CSID8610
1.94_127.0942m/z		127.0942	1.94	4-Silaspiro[3.4]octane	CSID24607729
1.95_155.0901m/z		155.0901	1.95	2-(3-Aminopropoxy)guanidine	CSID112164
1.97_615.3232m/z		615.3232	1.97	Moxaverine	CSID64045
2.08_357.2175m/z		357.2175	2.08	cyclizine lactate	CSID64553
2.09_101.0779m/z		101.0779	2.09	Silinane	CSID10757031
2.11_114.0917m/z		114.0917	2.11	Formylpiperidine	CSID16486
2.14_117.0413m/z		117.0413	2.14	(E)-Azodicarbonamide	CSID4575589
2.14_186.0643m/z		186.0643	2.14	1-Methoxymethyl-1H-benzotriazole	CSID472332
2.14_934.9852m/z		934.9852	2.14	2'-deoxycytidine-5'-triphosphate	CSID21232344
2.15_319.1790m/z		319.179	2.15	Ansatrienin A	CSID4445296
2.16_226.1406m/z		226.1406	2.16	Naluzotan	CSID9605731
2.17_185.1348m/z		185.1348	2.17	Carboprost	CSID4444532
2.21_261.1392m/z		261.1392	2.21	1,4-Dianilinobenzene	CSID6080
2.22_417.2493m/z		417.2493	2.22	Tetrahexosylceramide (d18:1/16:0)	CSID16744945
2.23_259.1624m/z		259.1624	2.23	Cucurbitacin L	CSID390403
2.27_903.9545m/z		903.9545	2.27	N-Isobutylhexacosanamide	CSID30777296

2.27_305.1751m/z	305.1751	2.27	prosolanapyrone II	CSID9997508
2.29_458.9294m/z	458.9294	2.29	Fipronil	CSID3235
2.35_381.2192m/z	381.2192	2.35	emodepside	CSID5293825
2.37_205.1078m/z	205.1078	2.37	(1E)-2-Methyl-1-butene-1-thiol	CSID30777485
2.37_143.0248m/z	143.0248	2.37	noxytiolin	CSID4418329
2.39_169.1414m/z	169.1414	2.39	6-Silaspiro[5.5]undecane	CSID14690870
2.39_197.1389m/z	197.1389	2.39	Muscarine	CSID8949
2.40_277.1636m/z	277.1636	2.4	Arbekacin	CSID61936
2.40_314.0498m/z	314.0498	2.4	ISAZOFOS	CSID35885
2.45_249.1344m/z	249.1344	2.45	HISTIDYLPROLINE DIKETOPIPERAZINE	CSID58646
2.51_186.0343m/z	186.0343	2.51	Phenethyl isothiocyanate	CSID15870
2.52_611.1945m/z	611.1945	2.52	Hesperidin	CSID10176
2.54_179.1578m/z	179.1578	2.54	(~2~H_4_)-1,2-Ethanediylbis(trimethylsilane)	CSID24607845
2.56_646.0317m/z	646.0317	2.56	Amiodarone	CSID2072
2.58_183.1164m/z	183.1164	2.58	Bibenzyl	CSID7364
2.59_396.2330m/z	396.233	2.59	Quinagolide	CSID2343034
2.61_560.3201m/z	560.3201	2.61	Auriculine	CSID391066
2.61_388.9380m/z	388.938	2.61	Fosfomycin calcium monohydrate	CSID10128527
2.61_836.4422m/z	836.4422	2.61	midecamycin	CSID4445365
2.62_390.0195m/z	390.0195	2.62	5-HYDROXYMETHYL MELOXICAM	CSID21896764
2.64_363.2112m/z	363.2112	2.64	1,1,4,4-TETRAPHENYLBUTANE	CSID120741
2.64_257.0633m/z	257.0633	2.64	1-Hydroxyhexane-1,2,6-tricarboxylate	CSID23283357
2.75_307.1891m/z	307.1891	2.75	Compactin diol lactone	CSID151560
2.76_168.0887m/z	168.0887	2.76	N-cyclopropylammeline	CSID4881949
2.77_310.1853m/z	310.1853	2.77	arg-leu	CSID295468
2.77_181.1073m/z	181.1073	2.77	Isobutanethiol	CSID10118
2.79_226.1545m/z	226.1545	2.79	Chaksine	CSID107747
2.79_271.0781m/z	271.0781	2.79	S-Methylcysteine	CSID196235
2.81_371.2372m/z	371.2372	2.81	artilide	CSID116301
2.81_294.0186m/z	294.0186	2.81	Pentrinitrol	CSID14614

2.81_407.2581m/z		407.2581	2.81	TESTOSTERONE PHENYLACETATE	CSID8106825
2.86_231.1248m/z		231.1248	2.86	Tirasemtiv	CSID30790768
2.91_439.1308m/z		439.1308	2.91	halopemide	CSID58938
2.91_595.1927m/z		595.1927	2.91	Tanaproget	CSID3572060
2.91_379.1049m/z		379.1049	2.91	thiotepa	CSID5254
2.94_525.2963m/z		525.2963	2.94	Kolanone	CSID30791168
2.94_433.2404m/z		433.2404	2.94	usambarensine	CSID4444762
2.95_474.9670m/z		474.967	2.95	Tilbroquinol	CSID59034
2.96_1229.1474m/z		1229.147	2.96	Lifitegrast	CSID10139520
2.99_179.0463m/z		179.0463	2.99	naphthal	CSID5960
2.99_604.3295m/z		604.3295	2.99	Salmeterol Xinafoate	CSID51216
3.01_127.0937m/z		127.0937	3.01	4-Silaspiro[3.4]octane	CSID24607729
3.01_192.1618n	192.1618	385.2617	3.01	Dodecanedinitrile	CSID70673
3.01_169.1424m/z		169.1424	3.01	Triethylenetetramine	CSID21106175
3.05_1190.1273m/z		1190.127	3.05	Glycol distearate	CSID55120
3.07_615.3665m/z		615.3665	3.07	(-)-Santiaguine	CSID30791163
3.07_239.1498m/z		239.1498	3.07	Pirimicarb	CSID29348
3.07_371.2414m/z		371.2414	3.07	THROMBOXANE B2	CSID4446261
3.09_475.2763m/z		475.2763	3.09	Amastatin	CSID388612
3.10_389.2453m/z		389.2453	3.1	12'-Apozeaxanthin	CSID9275794
3.10_407.2554m/z		407.2554	3.1	Estradiol enanthate	CSID19815
3.14_205.1783m/z		205.1783	3.14	Butylboronic acid	CSID19286
3.15_545.3148m/z		545.3148	3.15	Ladostigil	CSID181005
3.17_319.1876m/z		319.1876	3.17	(+)-10-Deoxymethynolide	CSID4445261
3.17_243.1466m/z		243.1466	3.17	prilocaine	CSID4737
3.19_204.1140m/z		204.114	3.19	Ampyrone	CSID2066
3.22_819.4471m/z		819.4471	3.22	Soyasaponin III	CSID10245008
3.26_430.2616m/z		430.2616	3.26	disobutamide	CSID61837
3.27_219.1407m/z		219.1407	3.27	3-(Methylthio)hexyl butanoate	CSID468337
3.27_742.4322m/z		742.4322	3.27	erythromycin C	CSID75735
3.28_493.2487m/z		493.2487	3.28	Vorapaxar	CSID8252668
3.31_351.2164m/z		351.2164	3.31	15-oxolipoxin A4	CSID27471351

3.31_360.2156m/z	360.2156	3.31 Fenpiverinium	CSID64566
3.31_266.1367m/z	266.1367	3.31 Tiglylcarnitine	CSID17216200
3.33_547.1718m/z	547.1718	3.33 Modafinil	CSID4088
3.37_565.1832m/z	565.1832	3.37 Agrostophyllin	CSID391049
3.37_427.1352m/z	427.1352	3.37 Droxidopa	CSID83927
3.41_453.2343m/z	453.2343	3.41 Trandolapril	CSID4588590
3.41_1129.0841m/z	1129.084	3.41 UDP-3-keto-alpha-D-glucose	CSID28533807
3.42_205.1747m/z	205.1747	3.42 (1,1-~2~H_2_)Silinane	CSID24607656
3.46_167.0921m/z	167.0921	3.46 1-Deoxy-L-mannitol	CSID392801
3.46_391.2437m/z	391.2437	3.46 Carboprost	CSID4444532
3.46_947.4947m/z	947.4947	3.46 Nafronyl oxalate	CSID17512
3.47_147.1208m/z	147.1208	3.47 n-Dibutyl sulfide	CSID10536
3.50_279.1598m/z	279.1598	3.5 Dibutyl phthalate	CSID13837319
3.52_195.1222m/z	195.1222	3.52 Acetylagmatine	CSID388732
3.52_614.3711m/z	614.3711	3.52 Indinavir	CSID4515036
3.54_292.1073m/z	292.1073	3.54 Ritalin hydrochloride	CSID8923
3.57_565.1883m/z	565.1883	3.57 Agrostophyllin	CSID391049
3.57_431.2552m/z	431.2552	3.57 Quingestanol acetate	CSID17136
3.62_273.2254m/z	273.2254	3.62 Trimethylsilyl laurate	CSID455029
3.63_231.1250m/z	231.125	3.63 Tirasemtiv	CSID30790768
3.65_484.3077m/z	484.3077	3.65 Lithocholic acid taurine conjugate	CSID388820
3.69_183.0746m/z	183.0746	3.69 Daminozide	CSID14593
3.70_169.1424m/z	169.1424	3.7 Triethylenetetramine	CSID21106175
3.73_163.1230m/z	163.123	3.73 2,5-Dihydropyridine	CSID10609350
3.73_193.0967m/z	193.0967	3.73 Acanthicifoline	CSID390929
3.73_497.2635m/z	497.2635	3.73 HISTIDYLPROLINE DIKETOPIPERAZINE	CSID58646
3.73_189.1018m/z	189.1018	3.73 Phenazone	CSID2121
3.73_949.5183m/z	949.5183	3.73 TRECETILIDE FUMARATE	CSID4945347
3.75_433.2695m/z	433.2695	3.75 Norethisterone enanthate	CSID199613
3.77_599.3405m/z	599.3405	3.77 Emixustat hydrochloride	CSID28529449
3.78_396.7474m/z	396.7474	3.78 immunomycin	CSID4445297
3.80_229.1662m/z	229.1662	3.8 4-Nitrosopiperidine	CSID30778640

3.80_973.5337m/z	973.5337	3.8 Prednisolone valerate acetate	CSID4447657
3.81_343.2469m/z	343.2469	3.81 PALMOXIRATE SODIUM	CSID2301081
3.82_219.9920m/z	219.992	3.82 4-Nitrophenyl hydrogen sulfate	CSID72581
3.83_341.1545m/z	341.1545	3.83 3-Biphenylol	CSID10903
3.83_303.0807m/z	303.0807	3.83 Methionylmethionine	CSID94154
3.83_652.6597m/z	652.6597	3.83 N-tetracosanoylsphinganine	CSID4446690
3.85_969.5758m/z	969.5758	3.85 Stigmatellin Y	CSID4445302
3.90_543.3270m/z	543.327	3.9 CUCURBITACIN P	CSID390404
3.90_535.1761m/z	535.1761	3.9 Propenidazole	CSID4950302
3.95_341.2089m/z	341.2089	3.95 Cyathin A3	CSID390575
4.00_724.3849m/z	724.3849	4 Kitasamycin	CSID4445382
4.01_229.1150m/z	229.115	4.01 Actinamine	CSID30791624
4.04_229.1671m/z	229.1671	4.04 dicyclohexylcarbodiimide	CSID10408
4.05_461.3110m/z	461.311	4.05 Dodecanedioic acid	CSID12213
4.07_1497.8070m/z	1497.807	4.07 Pfaffoside A	CSID30791205
4.08_595.2006m/z	595.2006	4.08 Neoponcirin	CSID16498764
4.09_271.0758m/z	271.0758	4.09 10-Oxabenzo(def)chrysen-9-one	CSID320641
4.09_433.1350m/z	433.135	4.09 aconitate F	CSID24606457
4.10_961.0148m/z	961.0148	4.1 Ponceau SX	CSID30790997
4.11_213.1008m/z	213.1008	4.11 (-)-Cytisine	CSID9818
4.11_230.1269m/z	230.1269	4.11 Arenaine	CSID2769416
4.11_455.2785m/z	455.2785	4.11 Latanoprost	CSID4470740
4.12_979.5573m/z	979.5573	4.12 isomigrastatin	CSID13083250
4.13_877.9670m/z	877.967	4.13 Triacontanol	CSID62194
4.15_449.1454m/z	449.1454	4.15 trinexapac	CSID10469309
4.16_1061.5870m/z	1061.587	4.16 gambiertoxin-4b	CSID10366292
4.16_321.1529m/z	321.1529	4.16 Pimelic acid	CSID376
4.17_966.0168m/z	966.0168	4.17 7,9-Dotriacontanediol	CSID35013339
4.21_725.4116m/z	725.4116	4.21 1,1,4,4-TETRAPHENYLBUTANE	CSID120741
4.22_379.2173m/z	379.2173	4.22 Cebranopadol	CSID29398942
4.22_1037.5866m/z	1037.587	4.22 Sativanine B	CSID4444916
4.23_183.0864m/z	183.0864	4.23 D-Sorbitol	CSID107748

4.27_585.3189m/z	585.3189	4.27	Lappaconitine	CSID390345
4.32_755.4338m/z	755.4338	4.32	Carmegliptin	CSID9592454
4.39_141.1100m/z	141.11	4.39	5-Silaspiro[4.4]nonane	CSID24607728
4.39_201.1344m/z	201.1344	4.39	Gyromitrin	CSID7827534
4.40_820.9592m/z	820.9592	4.4	Bisolvon	CSID4642584
4.40_1136.1228m/z	1136.123	4.4	C04977SRJ5	CSID4446686
4.40_653.3687m/z	653.3687	4.4	Etymemazine	CSID64847
4.40_645.3620m/z	645.362	4.4	taleranol	CSID20916
4.41_122.0730m/z	122.073	4.41	Bisphenol B	CSID59553
4.41_335.1733m/z	335.1733	4.41	lycaconitine	CSID390347
4.50_613.3725m/z	613.3725	4.5	Lasalocid A	CSID4514598
4.51_124.0885m/z	124.0885	4.51	geroquinol	CSID4445151
4.56_540.6350m/z	540.635	4.56	Hexachlorocyclopentadiene	CSID6233
4.56_335.1683m/z	335.1683	4.56	N-cyclopropylammeline	CSID4881949
4.59_872.9954m/z	872.9954	4.59	Technetium (99mTc) bicisate	CSID4805172
4.61_230.1271m/z	230.1271	4.61	Arenaine	CSID2769416
4.61_147.1203m/z	147.1203	4.61	n-Dibutyl sulfide	CSID10536
4.63_697.3964m/z	697.3964	4.63	(+)-Ingenol	CSID10282511
4.63_122.0729m/z	122.0729	4.63	Bisphenol B	CSID59553
4.63_357.1914m/z	357.1914	4.63	DIGITALIN	CSID390432
4.63_713.3756m/z	713.3756	4.63	Pibutidine	CSID4445602
4.63_1227.7615m/z	1227.762	4.63	Tetrahexosylceramide (d18:1/16:0)	CSID16744945
4.64_153.0913m/z	153.0913	4.64	prosolanapyrone II	CSID9997508
4.64_154.0866m/z	154.0866	4.64	Ruxolitinib	CSID25027389
4.67_229.1672m/z	229.1672	4.67	dicyclohexylcarbodiimide	CSID10408
4.67_505.3249m/z	505.3249	4.67	Dipyridamole	CSID2997
4.68_212.0520m/z	212.052	4.68	Sodium 4-Aminosalicylate Dihydrate	CSID4445547
4.69_872.9950m/z	872.995	4.69	Technetium (99mTc) bicisate	CSID4805172
4.71_738.4380m/z	738.438	4.71	2,2',3,3',4,5',6-HEPTABROMODIPHENYL ETHER	CSID30791500
4.71_217.0625m/z	217.0625	4.71	Anthrone	CSID6751
4.71_182.1122m/z	182.1122	4.71	Cortisol	CSID5551

4.71_264.0885m/z	264.0885	4.71 dimethylthiambutene	CSID10218
4.71_271.0784m/z	271.0784	4.71 mecysteine	CSID27113
4.72_490.2936m/z	490.2936	4.72 Buprenorphine	CSID559124
4.73_124.0887m/z	124.0887	4.73 geroquinol	CSID4445151
4.73_346.1889m/z	346.1889	4.73 LYSERGIDE, L-	CSID3843
4.75_315.0902m/z	315.0902	4.75 Sulfaphenazole	CSID5144
4.79_271.0778m/z	271.0778	4.79 S-Methylcysteine	CSID196235
4.81_659.3741m/z	659.3741	4.81 EUROPINE	CSID4575419
4.84_930.1864m/z	930.1864	4.84 3-Oxooctanoyl-CoA	CSID10140155
4.84_122.0731m/z	122.0731	4.84 Bisphenol B	CSID59553
4.84_208.1146m/z	208.1146	4.84 deacetylvindoline	CSID228679
4.84_125.0683m/z	125.0683	4.84 N-Nitrosodiethylamine	CSID5708
4.84_305.1735m/z	305.1735	4.84 prosolanapyrone II	CSID9997508
4.84_901.1801m/z	901.1801	4.84 Tedizolid Phosphate	CSID9651289
4.91_262.0638m/z	262.0638	4.91 Cicletanine	CSID49583
4.91_865.5116m/z	865.5116	4.91 Hydrocortisone butyrate (JP15/USP)	CSID24344
4.92_360.2159m/z	360.2159	4.92 Fenpiverinium	CSID64566
4.97_461.1184m/z	461.1184	4.97 Spiraprilat	CSID2298317
5.01_812.9635m/z	812.9635	5.01 Diclazuril	CSID401855
5.02_208.1150m/z	208.115	5.02 deacetylvindoline	CSID228679
5.02_801.4293m/z	801.4293	5.02 Ifenprodil tartrate	CSID570951
5.02_125.0682m/z	125.0682	5.02 N-Nitrosodiethylamine	CSID5708
5.03_122.0731m/z	122.0731	5.03 Bisphenol B	CSID59553
5.03_949.0142m/z	949.0142	5.03 DIGUANOSINE-PENTAPHOSPHATE	CSID4484327
5.03_1494.8246m/z	1494.825	5.03 Ganglioside GD3 (d18:1/18:0)	CSID16744913
5.03_163.1051m/z	163.1051	5.03 Levobupivacaine Hydrochloride	CSID105409
5.04_320.1093m/z	320.1093	5.04 Duloxetine	CSID54822
5.07_357.2030m/z	357.203	5.07 Prostaglandin B2	CSID4444404
5.08_641.0320m/z	641.032	5.08 Lonidamine	CSID36170
5.08_325.1274m/z	325.1274	5.08 Phe-his	CSID14892541
5.11_213.1054m/z	213.1054	5.11 albutoin	CSID2297355
5.11_230.1271m/z	230.1271	5.11 Arenaine	CSID2769416

5.11_627.3822m/z	627.3822	5.11 Heliotrine	CSID792587
5.18_341.1557m/z	341.1557	5.18 Fortimicin FU-10	CSID30791545
5.18_329.1566m/z	329.1566	5.18 S-methyl-L-methionine	CSID128519
5.20_122.0731m/z	122.0731	5.2 Bisphenol B	CSID59553
5.20_208.1145m/z	208.1145	5.2 deacetylvindoline	CSID228679
5.20_431.2207m/z	431.2207	5.2 Mexiletine hydrochloride	CSID20175
5.21_852.5132m/z	852.5132	5.21 alpha-chaconine	CSID391274
5.21_195.1326m/z	195.1326	5.21 Arcaine	CSID2141
5.21_125.0681m/z	125.0681	5.21 N-Nitrosodiethylamine	CSID5708
5.21_305.1736m/z	305.1736	5.21 prosolanapyrone II	CSID9997508
5.24_682.4273m/z	682.4273	5.24 Aridanin	CSID65922
5.24_245.1329m/z	245.1329	5.24 Triphenylmethane	CSID10169
5.28_344.2009m/z	344.2009	5.28 N,N-Didesmethyltamoxifen	CSID2300247
5.30_784.5289m/z	784.5289	5.3 IRGANOX 3114	CSID84061
5.30_222.1483m/z	222.1483	5.3 mazipredone	CSID5840
5.30_111.0783m/z	111.0783	5.3 pentanol	CSID6040
5.30_226.1062m/z	226.1062	5.3 Rhizocticin B	CSID30791558
5.30_168.1141m/z	168.1141	5.3 sinularin	CSID10469721
5.31_417.1770m/z	417.177	5.31 (-)-Brucine	CSID390579
5.38_195.1330m/z	195.133	5.38 Arcaine	CSID2141
5.38_208.1152m/z	208.1152	5.38 deacetylvindoline	CSID228679
5.38_770.9976m/z	770.9976	5.38 indisulam	CSID187608
5.38_153.0913m/z	153.0913	5.38 prosolanapyrone II	CSID9997508
5.42_399.2285m/z	399.2285	5.42 Pyrimethanil	CSID82753
5.43_186.1220m/z	186.122	5.43 artilide	CSID116301
5.43_229.1680m/z	229.168	5.43 dicyclohexylcarbodiimide	CSID10408
5.43_169.1426m/z	169.1426	5.43 Triethylenetetramine	CSID21106175
5.47_222.1494m/z	222.1494	5.47 mazipredone	CSID5840
5.47_168.1143m/z	168.1143	5.47 sinularin	CSID10469721
5.48_461.3131m/z	461.3131	5.48 Dodecanedioic acid	CSID12213
5.50_795.4618m/z	795.4618	5.5 Drotaverine	CSID1361582
5.53_195.1332m/z	195.1332	5.53 Arcaine	CSID2141

5.57_230.1272m/z	230.1272	5.57 Arenaine	CSID2769416
5.62_222.1487m/z	222.1487	5.62 mazipredone	CSID5840
5.67_937.0186m/z	937.0186	5.67 tartrazine acid	CSID56098
5.68_195.1332m/z	195.1332	5.68 Arcaine	CSID2141
5.72_255.0806m/z	255.0806	5.72 sulfadicramide	CSID7980
5.77_199.1529m/z	199.1529	5.77 (~2~H_5_)Phenol	CSID21170779
5.77_245.1279m/z	245.1279	5.77 (+)-Etomidate	CSID580864
5.77_414.2428m/z	414.2428	5.77 Astragaloside II	CSID30790840
5.77_146.1018m/z	146.1018	5.77 INOCOTERONE ACETATE	CSID5293176
5.77_583.3542m/z	583.3542	5.77 Perlapine	CSID15291
5.87_975.5084m/z	975.5084	5.87 Erythromycin stinoprate	CSID64401
5.92_352.1735m/z	352.1735	5.92 EUROPINE	CSID4575419
5.92_541.2990m/z	541.299	5.92 nostopeptolide A1	CSID8971518
5.95_682.4287m/z	682.4287	5.95 Aridanin	CSID65922
5.95_183.1168m/z	183.1168	5.95 Bibenzyl	CSID7364
5.95_344.2011m/z	344.2011	5.95 N,N-Didesmethyltamoxifen	CSID2300247
5.97_539.1449m/z	539.1449	5.97 Evoxanthidine	CSID391204
5.97_551.1265m/z	551.1265	5.97 Liriodenine	CSID9738
6.01_407.0962m/z	407.0962	6.01 Lancerin	CSID4444964
6.02_583.0340m/z	583.034	6.02 Brimonidine	CSID2341
6.05_715.0692m/z	715.0692	6.05 Chlorsulfuron	CSID43209
6.08_718.9345m/z	718.9345	6.08 Tetrakis(pentafluorophenyl)silane	CSID375930
6.09_1065.1773m/z	1065.177	6.09 dTDP-beta-D-oliose	CSID30791082
6.09_375.3965m/z	375.3965	6.09 Pentacosane	CSID11900
6.12_309.1037m/z	309.1037	6.12 Dexsotalol hydrochloride	CSID144864
6.14_1153.2344m/z	1153.234	6.14 Regrelor disodium	CSID9448186
6.14_551.1283m/z	551.1283	6.14 Sivelestat Sodium tetrahydrate	CSID139682
6.15_183.1139m/z	183.1139	6.15 cyclohexylbenzen	CSID12674
6.15_654.2009m/z	654.2009	6.15 Elagolix sodium	CSID23954980
6.15_472.2672m/z	472.2672	6.15 Erythrophleguine	CSID4444674
6.15_227.1155m/z	227.1155	6.15 METHYLCYTISINE	CSID204591
6.15_344.2005m/z	344.2005	6.15 N,N-Didesmethyltamoxifen	CSID2300247

6.15_583.3550m/z	583.355	6.15 Perlapine	CSID15291
6.17_524.6633m/z	524.6633	6.17 Hydroxyapatite	CSID14098
6.17_285.1115m/z	285.1115	6.17 Solvent Brown 1	CSID56367
6.18_1289.3250m/z	1289.325	6.18 MS15642725	CSID8502319
6.19_1185.7263m/z	1185.726	6.19 Agavoside A	CSID390452
6.19_195.1333m/z	195.1333	6.19 Arcaine	CSID2141
6.19_1039.1446m/z	1039.145	6.19 Cefminox	CSID64286
6.19_239.1618m/z	239.1618	6.19 Nonyl 2-hydroxypropanoate	CSID14190419
6.19_334.1751m/z	334.1751	6.19 Primidolol	CSID61835
6.22_371.2434m/z	371.2434	6.22 6-ketoprostaglandin f1?	CSID4444411
6.22_1304.3168m/z	1304.317	6.22 N-tetracosanoylsphinganine	CSID4446690
6.23_685.0696m/z	685.0696	6.23 boscalid	CSID184713
6.24_353.1397m/z	353.1397	6.24 3-O-methyl-8-prenylgalangin	CSID4445224
6.24_1329.7663m/z	1329.766	6.24 Phytolaccoside B	CSID390513
6.26_725.4462m/z	725.4462	6.26 Calpeptin	CSID66091
6.26_727.1240m/z	727.124	6.26 Cyclic pyranopterin monophosphate	CSID17221217
6.27_720.7950m/z	720.795	6.27 Clodronate Disodium	CSID21332554
6.27_539.1459m/z	539.1459	6.27 Evoxanthidine	CSID391204
6.27_701.1895m/z	701.1895	6.27 Sucrose octaacetate	CSID29073
6.28_167.0852m/z	167.0852	6.28 Fluorene	CSID6592
6.29_741.1434m/z	741.1434	6.29 flavodic acid	CSID64953
6.31_709.7087m/z	709.7087	6.31 Methyl Behenate	CSID12995
6.33_1241.2432m/z	1241.243	6.33 Cefditoren pivoxil	CSID4942398
6.33_285.1057m/z	285.1057	6.33 D-Ornaline	CSID388640
6.33_785.1669m/z	785.1669	6.33 PARECOXIB SODIUM	CSID7851185
6.34_940.0736m/z	940.0736	6.34 2,4-Dichlorobenzoyl-CoA	CSID7822078
6.34_837.1907m/z	837.1907	6.34 Juglanin	CSID10365524
6.35_829.1926m/z	829.1926	6.35 Apratastat	CSID9627567
6.36_961.0924m/z	961.0924	6.36 eugeniin	CSID391036
6.36_953.0909m/z	953.0909	6.36 Geraniin	CSID10270376
6.36_1223.7416m/z	1223.742	6.36 Zizyphine A	CSID4884461
6.37_859.2105m/z	859.2105	6.37 Besifloxacin hydrochloride	CSID8400086

6.37_651.0693m/z	651.0693	6.37	Urothion	CSID78342
6.39_697.1000m/z	697.1	6.39	2'-Inosinic acid	CSID18556963
6.39_525.0062m/z	525.0062	6.39	4-Bromobenzhydrol	CSID88691
6.39_891.0262m/z	891.0262	6.39	Bis(5'-guanosyl) tetraphosphate	CSID144813
6.39_725.4467m/z	725.4467	6.39	Calpeptin	CSID66091
6.39_866.1985m/z	866.1985	6.39	hexanoyl-CoA	CSID395736
6.39_844.9861m/z	844.9861	6.39	INDIGOTINDISULFONIC ACID	CSID4445584
6.39_325.1309m/z	325.1309	6.39	NICOFETAMIDE	CSID2298538
6.39_551.0071m/z	551.0071	6.39	sulfoglucobrassicin	CSID390219
6.39_383.2476m/z	383.2476	6.39	tetrofosmin	CSID4124
6.42_685.0967m/z	685.0967	6.42	Thiophanate-methyl	CSID2297683
6.44_707.1283m/z	707.1283	6.44	Glyparamide	CSID216134
6.44_828.9788m/z	828.9788	6.44	Isoconazole	CSID3629
6.46_916.2103m/z	916.2103	6.46	Octanoyl-coa	CSID393007
6.46_735.1284m/z	735.1284	6.46	sanguinarium chloride	CSID61894
6.47_325.2385m/z	325.2385	6.47	6-Decylubiquinol	CSID10128484
6.47_1300.2786m/z	1300.279	6.47	Ceramide (d18:1/24:0)	CSID4446684
6.47_749.1240m/z	749.124	6.47	Iguratimod	CSID110694
6.48_757.1559m/z	757.1559	6.48	Ritipenem acoxil hydrate	CSID10128319
6.52_417.1766m/z	417.1766	6.52	L-Kynurenine	CSID141580
6.52_1189.2265m/z	1189.227	6.52	losulazine hydrochloride	CSID49455
6.53_271.0753m/z	271.0753	6.53	10-Oxabenzo(def)chrysen-9-one	CSID320641
6.54_405.1219m/z	405.1219	6.54	2-cis,6-trans-farnesyl diphosphate	CSID393244
6.54_891.0248m/z	891.0248	6.54	Bis(5'-guanosyl) tetraphosphate	CSID144813
6.54_817.1754m/z	817.1754	6.54	Tetracenomycin E	CSID391881
6.55_841.1939m/z	841.1939	6.55	1,3,4,5-Tetracaffeoylquinic acid	CSID21864723
6.56_551.1510m/z	551.151	6.56	betanin	CSID10128200
6.56_795.1723m/z	795.1723	6.56	Faropenem medoxomil	CSID5293428
6.56_943.1982m/z	943.1982	6.56	Zeniplatin	CSID30791805
6.58_863.2262m/z	863.2262	6.58	clidinium bromide	CSID17942
6.58_1313.3253m/z	1313.325	6.58	Teniposide	CSID31930
6.60_741.7729m/z	741.7729	6.6	1,24-Tetracosanediol	CSID21121019

6.60_419.1121m/z	419.1121	6.6	AVERUFIN DIMETHYL ETHER	CSID35013930
6.60_737.1127m/z	737.1127	6.6	Diacerein	CSID24456
6.60_723.1159m/z	723.1159	6.6	Midazolam HCl	CSID39217
6.62_597.1618m/z	597.1618	6.62	mefenacet [JMAF]	CSID82816
6.65_252.2154m/z	252.2154	6.65	2-Hexaprenylphenol	CSID4444372
6.66_283.1250m/z	283.125	6.66	methaphenilene	CSID9884
6.67_733.1056m/z	733.1056	6.67	Malvidin Chloride	CSID62718
6.67_852.1809m/z	852.1809	6.67	Pentanoyl-CoA	CSID10140116
6.69_1129.1603m/z	1129.16	6.69	dTDP-D-glucose	CSID388455
6.70_513.1964m/z	513.1964	6.7	Phenylgalactoside	CSID92439
6.70_943.1998m/z	943.1998	6.7	Zeniplatin	CSID30791805
6.73_611.1597m/z	611.1597	6.73	Multinoside A	CSID10248307
6.76_885.2009m/z	885.2009	6.76	DARGLITAZONE SODIUM	CSID54853
6.77_904.8758m/z	904.8758	6.77	Fipronil Sulfone	CSID2336427
6.79_790.1406m/z	790.1406	6.79	Cefotiam hexetil hydrochloride	CSID153019
6.80_171.0303m/z	171.0303	6.8	p-Xylylenedithiol	CSID59434
6.83_219.1855m/z	219.1855	6.83	1,2,5-TRIMETHYLPYRROLE	CSID63445
6.84_237.0793m/z	237.0793	6.84	2-(4'-methylthio)butylmalic acid	CSID24808074
6.84_266.1397m/z	266.1397	6.84	Glycerol phenylbutyrate	CSID8657541
6.84_255.0978m/z	255.0978	6.84	N-(3,4-Dihydroxyphenyl)glutamine	CSID35032872
6.86_237.1606m/z	237.1606	6.86	Metabutethamine	CSID10644
6.87_286.9693m/z	286.9693	6.87	fenticlor	CSID7052
6.94_171.1011m/z	171.1011	6.94	1-(3-Furyl)-1,4-pentanediol	CSID19991118
6.94_1111.1239m/z	1111.124	6.94	4'-demethylrebeccamycin	CSID25052047
6.94_1248.2544m/z	1248.254	6.94	N-docosanoylsphinganine	CSID4446688
6.99_295.2408m/z	295.2408	6.99	neoabietadiene	CSID391686
7.01_1127.1685m/z	1127.169	7.01	Aclidinium bromide	CSID9694529
7.03_315.0704m/z	315.0704	7.03	1-Salicylate glucuronide	CSID2341148
7.03_1035.1282m/z	1035.128	7.03	Fenticonazole nitrate	CSID46839
7.04_231.2126m/z	231.2126	7.04	BUTYL BORATE	CSID12189
7.06_353.1122m/z	353.1122	7.06	Miraxanthin-III	CSID30791226
7.06_313.1781m/z	313.1781	7.06	octyl methoxycinnamate	CSID4511170

7.09_300.1738m/z	300.1738	7.09	Emixustat hydrochloride	CSID28529449
7.14_525.1277m/z	525.1277	7.14	5-Fluorouridine	CSID9056
7.14_183.0801m/z	183.0801	7.14	Carlina oxide	CSID144325
7.18_717.2161m/z	717.2161	7.18	Chryso-obtusin	CSID136879
7.19_609.1366m/z	609.1366	7.19	(3-Chlorophenyl)(9H-fluoren-2-yl)methanone	CSID2309061
7.20_401.2340m/z	401.234	7.2	Marinobufagenin	CSID10142870
7.20_209.1075m/z	209.1075	7.2	spironolactone	CSID5628
7.23_346.2713m/z	346.2713	7.23	Linoleoyl Ethanolamide	CSID4446566
7.23_930.0738m/z	930.0738	7.23	Tritriacontane	CSID11905
7.27_717.2315m/z	717.2315	7.27	Lactobionic acid	CSID7040
7.31_421.1177m/z	421.1177	7.31	caulerpin	CSID4481120
7.31_551.1333m/z	551.1333	7.31	Mahuannin D	CSID24842675
7.34_530.8284m/z	530.8284	7.34	Chromium(III) chloride hexahydrate	CSID94714
7.36_371.1376m/z	371.1376	7.36	alstonine	CSID390543
7.36_357.3012m/z	357.3012	7.36	Dibutyl tridecanedioate	CSID15276619
7.37_391.2798m/z	391.2798	7.37	Dolichotheline	CSID147033
7.48_191.1172m/z	191.1172	7.48	(-)-Cytisine	CSID9818
7.53_423.3755m/z	423.3755	7.53	Plakinamine A	CSID10243262
7.61_301.2533m/z	301.2533	7.61	allylestrenol	CSID205855
7.64_221.1621m/z	221.1621	7.64	CYCLURON	CSID15694
7.64_161.1406m/z	161.1406	7.64	methacholine	CSID1916
7.64_731.2107m/z	731.2107	7.64	sanggenon C	CSID390896
7.64_339.2838m/z	339.2838	7.64	SM(d18:0/14:0)	CSID24846886
7.66_322.2720m/z	322.272	7.66	3-ketosphinganine	CSID388895
7.66_209.0214m/z	209.0214	7.66	Angelicin	CSID10208
7.66_433.0798m/z	433.0798	7.66	Bensulfuron-methyl [ANSI, WSSA]	CSID49630
7.66_299.1981m/z	299.1981	7.66	Stearidonic acid	CSID4471933
7.69_662.1094m/z	662.1094	7.69	ELZASONAN CITRATE	CSID5005618
7.71_179.1581m/z	179.1581	7.71	(~2~H_4_)-1,2-Ethanediylbis(trimethylsilane)	CSID24607845
7.71_677.1196m/z	677.1196	7.71	lonapalene	CSID50751

7.78_275.1747m/z	275.1747	7.78 Allyxycarb	CSID21446
7.81_244.1292m/z	244.1292	7.81 Naluzotan hydrochloride	CSID28530504
7.82_429.2281m/z	429.2281	7.82 HARMALINE	CSID10211258
7.84_373.1496m/z	373.1496	7.84 beta-Syringin	CSID4475831
7.88_567.1677m/z	567.1677	7.88 Cucumopine	CSID390236
7.89_277.2285m/z	277.2285	7.89 Etidocaine	CSID34400
7.89_335.2332m/z	335.2332	7.89 fenalamide	CSID19480
7.89_295.2386m/z	295.2386	7.89 neoabietadiene	CSID391686
7.90_777.5134m/z	777.5134	7.9 rutamycin	CSID16735943
7.94_219.1828m/z	219.1828	7.94 12-Aminododecanenitrile	CSID11500520
7.94_309.1139m/z	309.1139	7.94 quinaldonitrile	CSID66650
7.97_518.1673m/z	518.1673	7.97 Nicomorphine	CSID4515048
7.98_183.1107m/z	183.1107	7.98 L-beta-homolysine	CSID2042257
7.98_311.1628m/z	311.1628	7.98 Nafenopin	CSID18456
7.98_220.0976m/z	220.0976	7.98 Radezolid	CSID9399462
7.98_329.1753m/z	329.1753	7.98 Raspberry ketone	CSID20347
7.99_209.2001m/z	209.2001	7.99 (2,2,6,6-~2~H_4_)Silinane	CSID24607714
7.99_387.2169m/z	387.2169	7.99 robustadial A	CSID140023
8.11_210.1592m/z	210.1592	8.11 Dimethirimol	CSID20014
8.28_335.2282m/z	335.2282	8.28 Fortimicin AP	CSID30791541
8.30_429.2647m/z	429.2647	8.3 Dicirenone	CSID8563314
8.30_232.1042m/z	232.1042	8.3 Mapracorat	CSID25104194
8.30_233.1105m/z	233.1105	8.3 renillafoulin A	CSID30791734
8.32_293.2253m/z	293.2253	8.32 3-Methyl-1-phenyl-2-butene	CSID19372
8.38_385.2359m/z	385.2359	8.38 Megestrol acetate	CSID11192
8.42_297.2570m/z	297.257	8.42 Ectocarpene	CSID28290131
8.46_292.1376m/z	292.1376	8.46 ambuphylline	CSID20536
8.48_327.0193m/z	327.0193	8.48 Diclofop	CSID35447
8.49_553.1396m/z	553.1396	8.49 (+)-Ketoconazole	CSID401695
8.62_324.2888m/z	324.2888	8.62 Linoleoyl Ethanolamide	CSID4446566
8.65_277.2282m/z	277.2282	8.65 Etidocaine	CSID34400
8.75_213.0674m/z	213.0674	8.75 (E)-butocarboxim	CSID4514630

8.75_329.1530m/z		329.153	8.75	tiapride	CSID5268
8.84_213.0683m/z		213.0683	8.84	Isobutyl 2-chlorobenzoate	CSID193012
9.32_421.2296m/z		421.2296	9.32	Levocabastine	CSID16736421
9.38_291.2076m/z		291.2076	9.38	4'-Hydroxyropivacaine	CSID30791783
9.39_271.2163m/z		271.2163	9.39	Levoamphetamine	CSID30477
9.67_428.2572n	428.2572	429.2645	9.67	Dicirenone	CSID8563314
9.69_327.0916m/z		327.0916	9.69	Vicine	CSID82575
9.73_320.2327m/z		320.2327	9.73	Dalcotidine	CSID114542
9.90_581.1705m/z		581.1705	9.9	Alatrofloxacin	CSID21243647
10.37_588.2603m/z		588.2603	10.37	Coelichelin	CSID2497530
10.37_537.2433m/z		537.2433	10.37	Pentostatin	CSID388759
10.38_591.2039m/z		591.2039	10.38	Cindunistat hydrochloride maleate	CSID30790737
10.40_497.2406m/z		497.2406	10.4	6-Hydroxymelatonin	CSID1794
10.40_246.1230m/z		246.123	10.4	Cyclohexyl 4-azidobenzoate	CSID24606652
10.40_376.1910m/z		376.191	10.4	Cyphenothrin	CSID35087
10.40_326.0905m/z		326.0905	10.4	Flumazenil	CSID3256
10.40_345.1136m/z		345.1136	10.4	Menadione	CSID3915
10.41_261.2789m/z		261.2789	10.41	3-Octanol	CSID11043
10.41_149.1027m/z		149.1027	10.41	Acetohydrazide	CSID13420
10.41_281.6549m/z		281.6549	10.41	Ergocornine	CSID66155
10.41_261.1447m/z		261.1447	10.41	L-gamma-Glutamyl-L-leucine	CSID133115
10.41_362.1168m/z		362.1168	10.41	Metampicillin	CSID5145919
10.41_234.0644m/z		234.0644	10.41	Milrinone	CSID4052
10.41_476.2562n	476.2562	477.2826	10.41	Voalla	CSID4447597
10.42_637.1709m/z		637.1709	10.42	Leiocarposide	CSID137456
10.42_353.1094m/z		353.1094	10.42	Miraxanthin-III	CSID30791226
10.45_605.2291m/z		605.2291	10.45	Oxantel pamoate	CSID4444539
10.46_262.0608m/z		262.0608	10.46	clomazone	CSID49469
10.46_524.2413m/z		524.2413	10.46	Diaplasinin	CSID4953370
10.65_391.2826m/z		391.2826	10.65	Dolichotheline	CSID147033
10.81_369.2389m/z		369.2389	10.81	Perindopril	CSID96956
10.86_291.2409m/z		291.2409	10.86	1-(12-Aminododecyl)-2-pyrrolidinone	CSID13595965

10.93_259.0747m/z	259.0747	10.93	Northienamycin	CSID116957
10.93_203.1153m/z	203.1153	10.93	Rilmenidine	CSID61963
10.93_262.1511m/z	262.1511	10.93	ser-arg	CSID8032215
11.05_402.3729m/z	402.3729	11.05	16,28-Secosolanidan-3-ol, (3.beta.,5.alpha.)-	CSID20042855
11.08_376.1316m/z	376.1316	11.08	Papaverine hydrochloride	CSID5859
11.08_300.0851m/z	300.0851	11.08	phosphopantothenic acid	CSID128
11.09_241.0848m/z	241.0848	11.09	Flavidin	CSID139512
11.56_281.0630m/z	281.063	11.56	2-Fluorobenzoic acid	CSID9547
11.56_207.0407m/z	207.0407	11.56	2-hydroxydibenzofuran	CSID59956
11.57_355.0861m/z	355.0861	11.57	Butamifos	CSID34329
11.94_209.1985m/z	209.1985	11.94	1-Methyl(2,2,5,5-~2~H_4_)silolane	CSID24607719
11.94_205.2039m/z	205.2039	11.94	3-Methyl(2,2,5,5-~2~H_4_)cyclopentanone	CSID24607197
11.95_177.1720m/z	177.172	11.95	(2,2,5,5-~2~H_4_)Cyclopentanone	CSID10430752
11.95_181.1668m/z	181.1668	11.95	1,1-diethyl-2-n-amylhydrazine	CSID486997
11.95_265.2649m/z	265.2649	11.95	3,5-Dimethyl-1-dodecylpyrazole	CSID498252
11.95_337.1934m/z	337.1934	11.95	Methylclostebol	CSID197559
11.95_271.0966m/z	271.0966	11.95	pinostrobin	CSID65961
12.06_355.0853m/z	355.0853	12.06	Butamifos	CSID34329
12.14_641.9554m/z	641.9554	12.14	Cefotetan disodium	CSID47903
12.25_825.8644m/z	825.8644	12.25	8,10-Heptacosanediol	CSID23255606
12.27_723.1630m/z	723.163	12.27	sparsomycin	CSID7822406
12.34_175.1562m/z	175.1562	12.34	Muscarine	CSID8949
12.37_439.3554m/z	439.3554	12.37	g-Tokoferol	CSID14266
12.37_289.2647m/z	289.2647	12.37	Tedisamil	CSID59237
12.41_269.1152m/z	269.1152	12.41	(-)-Irofulven	CSID130640
12.42_1221.3085m/z	1221.309	12.42	Multinoside A	CSID10248307
12.42_809.2066m/z	809.2066	12.42	Zopiclone N-oxide	CSID142713
12.44_187.1093m/z	187.1093	12.44	cis-Jasmone	CSID1266012
12.44_303.2307m/z	303.2307	12.44	Linoleic acid	CSID4444105

12.44_133.0967m/z	133.0967	12.44 L-Ornithine	CSID6026
12.49_803.0502m/z	803.0502	12.49 S-(1,2-Dichlorovinyl)glutathione	CSID4941923
12.50_223.0752m/z	223.0752	12.5 2-Methylanthraquinone	CSID6515
12.51_355.0862m/z	355.0862	12.51 Butamifos	CSID34329
12.51_429.1086m/z	429.1086	12.51 Penamecillin	CSID8426255
12.51_445.1439m/z	445.1439	12.51 thr-cys	CSID16568389
12.52_207.0421m/z	207.0421	12.52 2-hydroxydibenzofuran	CSID59956
12.54_737.1919m/z	737.1919	12.54 ketazolam	CSID31110
12.54_653.0920m/z	653.092	12.54 Prebetanin	CSID4885085
12.58_581.0440m/z	581.044	12.58 UDP-alpha-D-glucuronic acid	CSID16522
12.69_353.3206m/z	353.3206	12.69 Heptylbenzene	CSID13492
12.72_922.1677m/z	922.1677	12.72 2-Naphthoyl-CoA	CSID10138057
12.73_878.1232m/z	878.1232	12.73 5-hydroxy-2-furoyl-CoA	CSID21864787
12.75_717.5103m/z	717.5103	12.75 pregnenolone acetate	CSID2005918
12.77_1135.7636m/z	1135.764	12.77 5-O-Mycaminosyprotylonolide	CSID10278511
12.86_177.1733m/z	177.1733	12.86 (2,2,5,5-~2~H_4_)Cyclopentanone	CSID10430752
12.86_209.2002m/z	209.2002	12.86 1-Methyl(2,2,5,5-~2~H_4_)silolane	CSID24607719
12.86_139.1200m/z	139.12	12.86 3-Methyl-1,5-pentanediamine	CSID10645088
12.86_167.1545m/z	167.1545	12.86 3-Methyl-2,3-dihydro-1H-pyrrole	CSID14172782
12.86_223.2160m/z	223.216	12.86 Heptanonitril	CSID11866
12.86_153.1367m/z	153.1367	12.86 Heptylhydrazine	CSID16608
12.92_439.3567m/z	439.3567	12.92 13,28-Epoxyurs-11-en-3-one	CSID35013781
12.96_263.2495m/z	263.2495	12.96 1-Methyl-1-(~2~H_3_)methylsilinane	CSID24607660
12.99_626.0870m/z	626.087	12.99 (R)-afoxolaner	CSID30790744
12.99_795.2235m/z	795.2235	12.99 Rivoglitazone	CSID2316729
13.00_597.0706m/z	597.0706	13 diquat dibromide	CSID25613
13.01_1127.3151m/z	1127.315	13.01 Daunorubicin hydrochloride	CSID56512
13.01_663.1460m/z	663.146	13.01 Deoxyadenosine monophosphate	CSID12079
13.01_1245.3901m/z	1245.39	13.01 Pectolinarin	CSID147700

13.02_713.1693m/z	713.1693	13.02	Penicillin G Sodium Salt	CSID6014
13.03_1025.2513m/z	1025.251	13.03	Temocapril hydrochloride	CSID391963
13.04_677.4552m/z	677.4552	13.04	Ganglioside GA1 (d18:1/25:0)	CSID17216301
13.04_751.2065m/z	751.2065	13.04	Parishin B	CSID23551088
13.06_1243.8552m/z	1243.855	13.06	Diethanolamine fusidate	CSID30791487
13.06_633.4240m/z	633.424	13.06	Ganglioside GM3 (d18:1/24:0)	CSID16744868
13.06_825.0797m/z	825.0797	13.06	mallotinic acid	CSID8231698
13.06_327.0918m/z	327.0918	13.06	Vicine	CSID82575
13.09_355.0865m/z	355.0865	13.09	Butamifos	CSID34329
13.16_351.2255m/z	351.2255	13.16	Fucoxanthin acetate	CSID24607918
13.21_441.2672m/z	441.2672	13.21	15-Crown-5	CSID33416
13.25_675.4127m/z	675.4127	13.25	Danazol	CSID26436
13.27_243.1556m/z	243.1556	13.27	Tris(hydroxymethyl)aminomethane	CSID6257
13.31_679.4464m/z	679.4464	13.31	(-)-noracymethadol	CSID119159
13.33_763.5353m/z	763.5353	13.33	sphinganine 1-phosphate	CSID559277
13.36_613.3738m/z	613.3738	13.36	Compactin diol lactone	CSID151560
13.40_849.5757m/z	849.5757	13.4	alpha-Phocaecholic acid	CSID167853
13.40_631.4610m/z	631.461	13.4	Zinc stearate	CSID10705
13.42_812.6350m/z	812.635	13.42	Bromosulfophthalein	CSID5152
13.43_784.6147m/z	784.6147	13.43	2,3,3',4,4',5,5'-PCB	CSID35108
13.43_745.0434m/z	745.0434	13.43	Clazuril	CSID53093
13.45_761.5215m/z	761.5215	13.45	Enisoprost	CSID4470738
13.47_251.0581m/z	251.0581	13.47	9,10-DICYANOANTHRACENE	CSID64194
13.47_327.0942m/z	327.0942	13.47	INDOLEACETYL GLUTAMIC ACID	CSID28567151
13.48_339.2000m/z	339.2	13.48	Atrazine-desethyl-2-hydroxy	CSID96906
13.50_673.4670m/z	673.467	13.5	Hepoxilin A3	CSID4446332
13.53_1151.3240m/z	1151.324	13.53	Novclobiocin 105	CSID30791079
13.54_1107.2932m/z	1107.293	13.54	lodoxamide tromethamine	CSID9367206

13.55_1283.4127m/z	1283.413	13.55	Cyclothialidine	CSID391757
13.58_743.1902m/z	743.1902	13.58	Ampicillin sodium	CSID389889
13.58_680.9971m/z	680.9971	13.58	Fosfructose	CSID150604
13.58_437.3439m/z	437.3439	13.58	Methyl 2-[(trimethylsilyl)oxy]icosanoate	CSID457355
13.59_1179.3556m/z	1179.356	13.59	Novclobiocin 104	CSID30791078
13.60_665.1210m/z	665.121	13.6	Sulfadimethoxine Sodium	CSID64667
13.63_421.3479m/z	421.3479	13.63	Trimethylsilyl 19-methylicosanoate	CSID24606304
13.67_1485.5444m/z	1485.544	13.67	(-)-syringaresinol O,O'-bis(beta-D-glucoside)	CSID391147
13.70_855.1284m/z	855.1284	13.7	cefpodoxime	CSID4514925
13.73_668.7898m/z	668.7898	13.73	Chlorethoxyfos	CSID82758
13.73_351.2230m/z	351.223	13.73	Fucoxanthin acetate	CSID24607918
13.73_741.1836m/z	741.1836	13.73	Kandelin A2	CSID391043
13.75_321.1456m/z	321.1456	13.75	Auraptene	CSID1267148
13.75_501.0815m/z	501.0815	13.75	Demeclocycline hydrochloride	CSID10482189
13.75_645.0726m/z	645.0726	13.75	Digallic acid	CSID334
13.75_621.7248m/z	621.7248	13.75	Docosane	CSID11899
13.75_813.1882m/z	813.1882	13.75	Lancerin	CSID4444964
13.75_229.9084m/z	229.9084	13.75	Nitrapyrin	CSID15205
13.75_100.9636m/z	100.9636	13.75	Perchloric acid	CSID22669
13.79_130.0161m/z	130.0161	13.79	3-Amino-6-chloropyridazine	CSID20342
13.83_196.8981m/z	196.8981	13.83	Halothane	CSID3441
13.92_262.8707m/z	262.8707	13.92	Diammonium tetrathioxomolybdate(2-)	CSID21251162
